# High fat diet-induced loss of pituitary plasticity in aging female mice with ablated leptin signaling in somatotropes

**DOI:** 10.3389/fendo.2025.1617109

**Published:** 2025-07-16

**Authors:** Tiffany K. Miles, Angela K. Odle, Stephanie D. Byrum, Alex N. Lagasse, Anessa C. Haney, Victoria G. Ortega, Ashley K. Herdman, Melanie C. MacNicol, Angus M. MacNicol, Gwen V. Childs

**Affiliations:** ^1^ Department of Neuroscience, University of Arkansas for Medical Sciences, Little Rock, AR, United States; ^2^ Department of Biochemistry and Molecular Biology, University of Arkansas for Medical Sciences, Little Rock, AR, United States; ^3^ Department of Biomedical Informatics, University of Arkansas for Medical Sciences, Little Rock, AR, United States; ^4^ Arkansas Children's Research Institute, University of Arkansas for Medical Sciences, Little Rock, AR, United States

**Keywords:** pituitary, scRNA-seq, high-fat diet, obesity, leptin, LEPR, aging

## Abstract

**Introduction:**

Somatotropes lacking leptin receptors (LEPR) produce less growth hormone and are poorly responsive to growth hormone releasing hormone (GHRH). Transcriptomic analysis reveals that the mutant somatotropes contain progenitor cell markers (Sox9+) and multiple pituitary hormone transcripts-(*Pomc*, *Prl*, *Lhb*, *Tshb* and *Cga*), suggesting that the cells are progenitor cells. The resulting GH deficiency contributes to adult-onset obesity in the mutant, due to an increase in abdominal fat.

**Objectives:**

This study examined how a high-fat diet (HFD) affected pituitary transcriptomic function in older (10-month) female mutants lacking leptin receptors (LEPR) in somatotropes and intact littermate controls. We hypothesized that pituitary cells from both the older control females and the female mutants would be greatly affected by the oxidative stress from the HFD.

**Methods:**

Mice were exposed to a 60% HFD for 16 weeks, followed by glucose tolerance testing and 3-day monitoring in metabolic cages (CLAMS). Pituitaries were harvested, cells dispersed and subjected to single cell-RNA-seq (scRNA-seq) with bioinformatic analysis. Serum was collected and analyzed for pituitary hormones and cytokines.

**Results:**

The HFD resulted in elevated serum leptin and IL-6 in both mutants and controls, and reduced serum growth hormone (GH) and prolactin (PRL) levels. However, adrenocorticotropin (ACTH) levels were elevated in controls but not mutants. Unexpectedly, whereas controls gained as much weight as younger females, somatotrope LEPR-null mutants on a HFD gained only 75% of the weight of controls, were more glucose tolerant, consumed less food, were more active in the metabolic cages, and had lower serum levels of insulin. Analysis of scRNA-seq revealed that the HFD induced differentially expressed genes (DEGs) in more distinct pituitary cell populations of older mice compared to previously reported findings in younger control females, indicating greater vulnerability in the older pituitary population. This was especially true in the mutant pituitary population. Ingenuity Pathway Analysis indicated that the DEGs included targets of critical upstream regulators important for pituitary cell function and plasticity (CREB, Fox01, cAMP, STAT3, insulin, TRH, GnRH, and leptin signaling pathways), with most pathways predicted to be downregulated by the HFD. Unlike controls, HFD-fed mutant cell populations exhibited DEGs consistent with the downregulation of translational regulatory pathways. Notably, the HFD reversed the increased expression of progenitor cell markers (*Sox9*+) and multiple pituitary hormone transcripts seen in the mutant on a control diet. Similarly, the HFD also reversed the expression of multiple pituitary hormone transcripts and progenitor markers in lactotropes, thyrotropes, and corticotropes from mutants.

**Conclusion:**

The findings supported our hypothesis that both aging and the mutation (loss of LEPR in somatotropes) would render these mice more sensitive to a HFD as more pituitary cell types were affected transcriptionally. Collectively, these findings indicate that HFD and/or obese state may compromise pituitary plasticity by down-regulating translational processes and reducing expression in cells that may have multipotential functions. The oxidative stress of a HFD may thus limit the expression of pituitary progenitor cells.

## 1 Introduction

The anterior pituitary is noted for its ability to adapt and optimize its cell population to the changing needs of the body. Its specialized hormone-secreting cells include somatotropes that produce and secrete growth hormone (GH); gonadotropes, which produce the gonadotropins—luteinizing hormone (LH) and follicle-stimulating hormone (FSH); corticotropes, which produce adrenocorticotropin (ACTH); thyrotropes, which produce thyroid-stimulating hormone (TSH); and lactotropes, which produce prolactin (Prl). Distinct mature cell populations initially develop along lineages governed by unique transcription factors and signaling pathways (1–3). However, some immature stem and progenitor cells remain in the adult pituitary. These immature cells are expanded following ablation or injury of the pituitary (4, 5) or loss of the target organ (6–8). They are often characterized by the presence of more than one pituitary hormone (5, 8), indicating that they may be multipotential. Such cells may contribute to the pituitary plasticity that facilitates responses to various physiological and pathological conditions (8–10).

Somatotropes are the most numerous among all pituitary cell populations. They secrete growth hormone (GH) in response to growth hormone-releasing hormone (GHRH) from the hypothalamus and function as metabolic sensors that receive signals from peripheral organs and serum nutrients. Adipose tissue is particularly vital for signaling metabolic status to somatotropes, primarily through the adipokine leptin (LEP), which communicates via the leptin receptor (LEPR) and the JAK/STAT signaling pathway (11).

First discovered as an anorexigenic hormone, leptin signals satiety and fat reserves not only to the hypothalamus, but also directly to pituitary somatotropes and other pituitary cell populations (among other tissues), thereby informing them of the body's metabolic and nutritional state (12). Typically, leptin signaling induces the maturation of somatotropes (13–16). However, the effects of hyperleptinemia due to a high-fat diet (HFD)-induced obese condition are not well understood. Obesity associated hyperleptinemia suppresses somatotrope function in both humans and rodents (17–23), resulting in decreased GH synthesis and release and reduced receptors for GHRH (GHRHR) and ghrelin [GHS-R] (24–27).

Our recent studies of young adult mice on a high-fat diet (HFD) utilized single-cell RNA-sequencing transcriptomics analysis (scRNA-seq) to assess the impact of diet-induced obesity on the transcriptomes of somatotropes and other pituitary cell types (28). This study indicated that lactotropes and stem cells in females were particularly vulnerable to the HFD, exhibiting signs of oxidative stress and mitochondrial dysfunction. While the HFD caused reduced serum growth hormone (GH), mRNA levels for *Gh* declined only in the lactotrope population. This multihormonal lactosomatotrope population was particularly sensitive to the HFD, suggesting that pituitary plasticity is a metabolically vulnerable process. This is translationally significant as it suggests that a HFD could hinder the transdifferentiation of one pituitary cell population to support the function of another population.

We designed the next phase of this study to focus on the impact of a high-fat diet (HFD) on the pituitary transcriptome of mutant females bearing somatotropes that lack the leptin receptor (somatotrope LEPR-null). Additionally, we initiated the study with 6-month-old female controls and mutants because the phenotype of the mutant was more pronounced in this age group (11, 14, 15, 29–32). We were also particularly interested in these somatotrope LEPR-null mutants because our recent study showed that, on a control diet, the somatotropes expressed multiple non-somatotrope hormone transcripts, indicating enhanced multihormonal expression. They also exhibited an immature gene signature similar to that of progenitor cells (13), including the SRY-related HMG box transcription factor (Sox9+) marker for differentiating progenitor cells (7, 8). Exposure to the HFD provided us with an opportunity to determine the impact of diet and obesity on mutant somatotropes with this immature signature. In addition, we report new findings showing multihormonal expression in lactotropes, thyrotropes, and corticotropes from the mutant females. These mice thus provided a cellular model to test the impact of a HFD on multihormonal expression and pituitary plasticity.

Here, we report the effects of a high-fat diet (HFD) on multiple pituitary populations from aging females who were 10 months old at the end of the study, which is considered middle age (33) and other researchers (34–36). The aging pituitary cell populations in the control group appeared more vulnerable to the oxidative stress caused by obesity than those in younger populations as the HFD impacted more cell populations compared to just two cell populations in the young females (28). This study also provides new insights into the effects of LEPR loss in somatotropes (13). We will also demonstrate that the HFD reduced the expression of the multihormonal/progenitor cell gene profile in somatotropes, lactotropes, thyrotropes, and corticotropes. These reductions may indicate stimulated differentiation or the heightened vulnerability of progenitor/stem cells to the oxidative stress associated with obesity. The latter hypothesis suggests that a high-fat diet (HFD) might compromise pituitary plasticity.

## 2 Materials and methods

### 2.1 Handling of animals

The Institutional Animal Care and Use Committee approved all experimental procedures (letter attached). The FVB hybrid strain of mice was FVB.129P2-Pde6b + Tyr c-ch/AntJ, which had also been used previously (28). The female deletion mutant mice carried one Cre-recombinase allele driven by the rat GH promoter and two alleles of floxed LEPR-exon 1. Littermate controls carried two alleles of floxed LEPR. We bred the mutants in cages containing one male expressing Cre-GH and floxed LEPR and two females expressing only floxed LEPR. At six months, the mutant females cycled irregularly and were poor breeders, which limited our animal numbers. Due to these limitations, we could not collect animals from the same cycle stage.

The HFD (fat, 60 kcal%; carbohydrate, 36 kcal%; protein, 19 kcal%) consisted of palatable pellets containing lard and soybean oil as sources of fat (ENVIGO, TD.06414). The control diet (CD) was the ENVIGO Reduced Sucrose Control Diet (10% fat, TD.08806). Starting at 6 months of age, five to nine mice per genotype (Control vs. Mutant) were fed either the HFD (n=5 control, n=7 mutant) or the CD (n=5 control, n=9 mutant) for 16 weeks. Cells from five to six females from each of these groups provided pituitary cells for the single-cell RNA sequencing (scRNA-seq), with each pool containing cells from two to three females, as described previously (28, 30).

Parallel studies were conducted on male mice, comparing the effects of LEPR loss in somatotropes and exposure to a high-fat diet (HFD). Transcriptomic analysis of these male pituitary cells revealed more modest changes in expression after the ablation of LEPR in somatotropes, and no effects of the HFD on weight gain or pituitary transcripts were observed. Since further studies of additional males are needed to explore this sex difference, data from the males will not be included in this report.

### 2.2 Thermoneutral housing

Mice were housed at 28.4°C (thermoneutrality) on an 11-hour light cycle (starting at 07:00) and a 13-hour dark cycle (starting at 18:00). Before exposure to the high-fat diet (HFD) or control diet (CD) for 16 weeks, we acclimated both mutant and control mice in the thermoneutral environment for one week. The dedicated thermoneutral room was retrofitted to maintain a temperature of 28.4°C, which is within the recommended range for most mouse strains. We ensured that the temperature inside the mouse cages did not exceed the recommended levels and monitored the room temperature remotely using a Room Alert device from AVTECH Software, Inc. Recent studies discussing its translational applicability to human disease (37–47) informed and supported our decision to use a thermoneutral environment. We monitored room temperature, humidity, and animal health status daily, and recorded cage changes, food intake, and weight measurements weekly.

### 2.3 Glucose tolerance tests

One week before the tests in the metabolic cages and euthanasia, we conducted glucose tolerance tests (GTT) as previously described (28). Briefly, after determining the basal glycemia levels from a tail snip, mice received an intraperitoneal injection of 1 g/kg of 20% glucose. Tail snips were utilized to collect small blood samples for glycemia assessment at 15, 30-, 60-, 90-, and 120-minutes post-injection. Mice were then returned to their cages to acclimate for one week before entering the metabolic cages.

### 2.4 Metabolic cage tests

One week prior to euthanasia, the mice were transferred to the Comprehensive Lab Animal Monitoring System (Oxymax CLAMS, Columbus Instruments) to assess their metabolic health, as described in previous studies (14, 15, 29). Water and a powdered version of their diet were available during their time in the CLAMS unit. Food and water were available ad libitum. The metabolic cages were in the same thermoneutral room where the mice were housed (see above). As described previously, activity, food intake, and oxygen and CO2 consumption were measured over a period of 36 hours.

### 2.5 Euthanasia

On the same morning that the mice were removed from the CLAMS unit, they were euthanized for sample collection. The mice were first deeply anesthetized with isoflurane and then decapitated

by guillotine. Pituitaries were removed for dispersion and single-cell RNA-seq, and serum was collected for hormone and cytokine analyses.

### 2.6 Pituitary cell dispersion

As previously reported, we conducted scRNA-seq on cells from five-six (6) female pituitaries per experimental group [(control or mutant, CD or HFD) (13, 28)]. The dispersion protocol was optimized for mouse pituitaries based on the method previously reported for rats (48). We reduced the concentration of trypsin from 5 to 3 mg/ml and incubated the pituitary pieces for 20 min at 37 C. After centrifugation for 10 min (170 g), the supernatant was replaced with dispersion solution containing trypsin inhibitor and DNase, and the cells were gently dispersed through an 18-gauge needle 15–20 times. After dispersion and centrifugation, the cells were gently washed and resuspended in DMEM without gentamicin, with further trituration as needed to ensure a single-cell suspension. Individual pituitary cell suspensions were then pooled within treatment groups (2–3 pituitaries per pool) for counting, and the cell concentration within each pool was adjusted to target 10,000 cells optimally, according to 10× Genomics guidelines.

### 2.7 Single-cell RNA sequencing

Samples were submitted to the UAMS Cancer Institute Genomics Core facility for scRNA-seq. Visual cell debris or aggregates were removed by passing cell suspensions through a Flowmi (SP BEL-ART, catalog <ns/> 136800040) cell strainer (40 µm). After centrifuging cells at 250g for 10 minutes, they were resuspended in 1× phosphate-buffered saline (Ca2+/Mg2+ free) containing 0.04% (w/v) bovine serum albumin. To determine cell concentration and viability, cells were stained with ReadyProbes_Cell Viability Imaging Kit, Blue/Green (Thermo Fisher Scientific, catalog <ns/> R37609), and manually counted under a microscope (EVOS M7000, Thermo Fisher Scientific)

All protocols for single-cell library generation and bioinformatics analysis were performed as previously described (13, 28). Single-cell 3′ library generation utilized a 10Å~ Genomics Chromium Controller and Chromium Next GEM Single Cell 3′ Reagent Kits v3.1 (Dual index). We adhered to the manufacturer's protocol. Cell suspensions were loaded onto the middle four channels of a Chromium Single-Cell G chip, following the manufacturer's instructions, with the aim of achieving 8000 to 10,000 cells per channel. After generating single-cell gel beads in emulsions, we conducted reverse transcription, fragmentation, cDNA amplification, library preparation, and barcoding. We assessed the concentration and size distribution of the libraries using a Fragment Analyzer and a Qubit fluorometer. Libraries were sequenced on the Illumina NovaSeq 6000 platform in paired-end mode (read 1: 28 cycles, read 2: 90 cycles, i7: 10). This generated a minimum of 20,000 read pairs per cell. 10Å~ Genomics Cell Ranger 7.1 mkfastq wrapper was used to perform sample demultiplexing and generate fastq files.

### 2.8 Bioinformatic analyses

Our previous studies have described quality control, bioinformatics clustering and differential gene expression (11, 13, 28). We analyzed demultiplexed fastq files generated by the UAMS Genomics Core with the 10Å~ Genomics Cell Ranger 3.1.0 count function for sequence alignment and gene counting, a self-contained single-cell RNAseq pipeline developed by 10Å~ Genomics. We aligned the reads to the University of California, Santa Cruz (UCSC) mm10 reference transcriptome using STAR and transcript counts were generated (49, 50).

We used the R package Seurat to further analyze the filtered, aggregated, and depth normalized counts generated by cellranger count and cellranger aggr (http://software.10xgenomics.com/single-cell/overview/welcome) (51). Cells with unique feature counts over more the 75th percentile plus 1.5 times the interquartile range (IQR) or less 200 unique features, total gene counts more than more the 75th percentile plus 1.5 times the IQR or less than 1000 counts, and/or mitochondrial feature percentage more the 75th percentile plus 1.5 times the IQR or less than the 25th percentile minus 1.5 times the IQR were filtered out of the data. Next, the 2000 highest variable features were selected. The data were then scaled by a linear transformation and variation associated with cell death and cell cycling was regressed out of the scaled data using mitochondrial feature percentage and cell cycle scoring values. We normalized counts using the SCTransform and PCA was performed on the scaled data.

We used the Uniform Manifold Approximation and Projection (UMAP) to visualize and explore the clustering results. Seurat's FindNeighbors and FindClusters functions were optimized to label clusters based on the visual clustering in the projections. Seurat FindAllMarkers function was used to identify gene markers that define cluster cell types. We used known markers of expected pituitary cell types (52, 53) to assign appropriate cell type labels (54). Differential expression analysis was performed using the FindMarkers to compare similar cell types across diet and gender. We used the MAST test, a GLM framework that treats cellular detection rate as a covariate, to determine statistically different transcription (55). Genes with an false discovery rate adjusted P <.055 and a Log2FC ≥ 0.58 (fold increase 1.5) were statistically significant.

### 2.9 Multiplex assays

Multiplex mouse adipokine or pituitary hormone enzyme immunoassays (Millipore Sigma) (14, 29) were used to quantify pituitary hormones, adipokines, and inflammatory markers as follows: The pituitary hormone EIA kit (MPTMAG-49K-05), RRID: AB_2811194, was used to quantify adrenocorticotropin-ACTH; luteinizing hormone-LH; follicle stimulating hormone-FSH; thyroid stimulating hormone-TSH; and prolactin-PRL. The GH kit-RPTMAG-86K-01 (RRID: AB_2716840) was used for GH quantification. The mouse adipokine kit quantified serum levels of adiponectin, interleukin-6 (IL-6), insulin, leptin, monocyte chemoattractant protein-1 (MCP-1), plasminogen activator inhibitor-1 (PAI-1), and tumor necrosis factor-alpha (TNF-α), and resistin (MADKMAG-71K-07, RRID: AB_2801416). We assayed leptin by the Quantikine Elisa Systems, MOB00B (R&D systems, RRID: AB_2943468). All multiplex assays were run on a Luminex LX200 instrument. We validated all assays used in this study in previous reports (11, 14, 15, 28, 30, 32, 56).

### 2.10 Statistics and power analyses

*Post hoc*power analyses determined the number of replicates using the G* Power application. *https://www.psychologie.hhu.de/arbeitsgruppen/allgemeine-psychologie-und-arbeitspsychologie/gpower.* We employed an F-test for population variance executed in R code. GraphPad PRISM (10) was utilized with p<0.05 and tests suitable for the design [Example: 2-way ANOVA with Bonferroni *post hoc* tests].

## 3 Results

### 3.1 Weight gain and glucose tolerance

**Figure 1:**
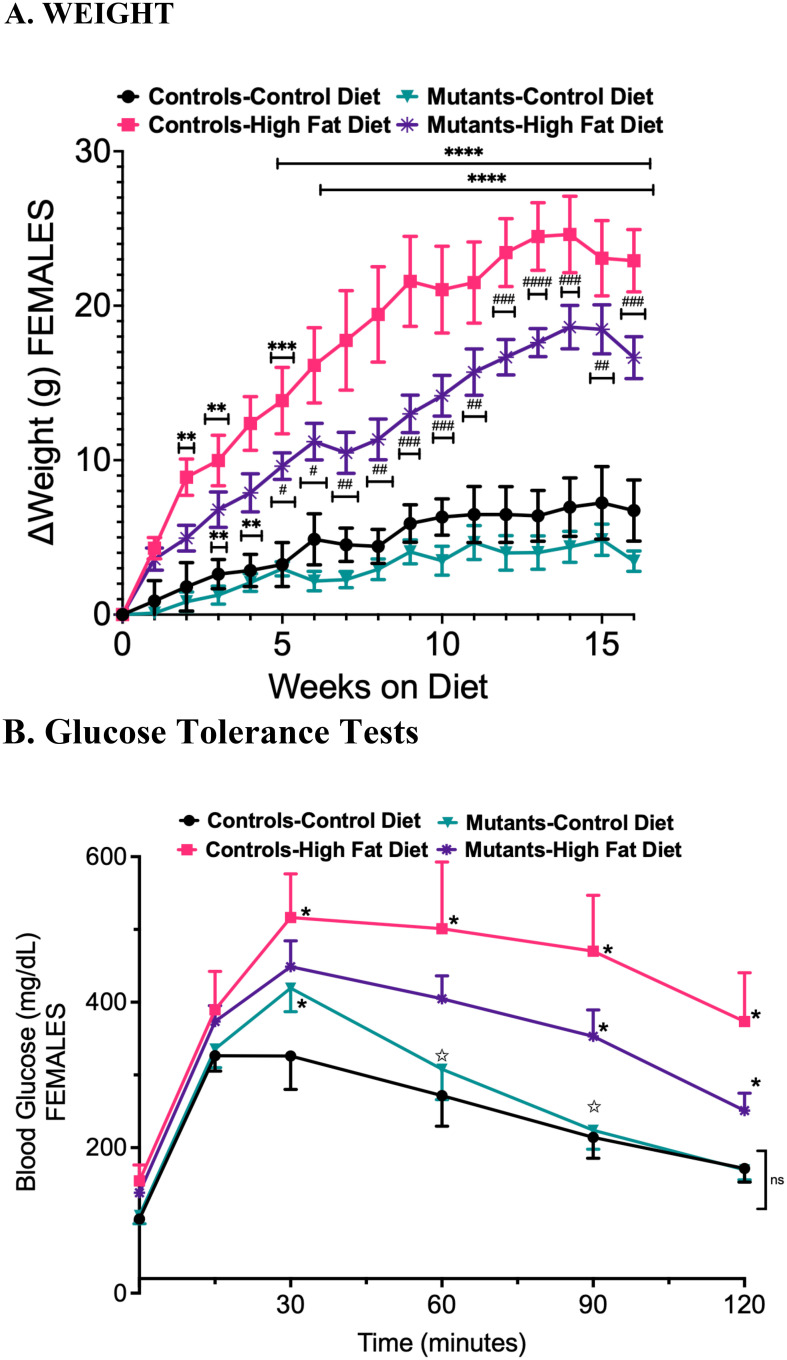
**(A)** Weight gain after 16 weeks on the high-fat diet. Mutants bear somatotropes that lack LEPR. **(B)** Glucose Tolerance Tests compare controls and mutants (LEPR-null somatotropes), and stars indicate significance. Stars indicate significant difference comparing controls on a control diet with controls on a HFD, two-way ANOVA and Tukey's test; number of stars indicates p value (***=p<0.001). Stars on the mutant HFD graph show difference from both groups on a control diet. Hashtags (#) show the difference between the Controls and Mutants on a HFD with the number of hashtags equal to the p value. (###=p<0.001) In **(B)**, closed stars indicate significant difference compared to controls on a control diet. Open stars show difference between mutants on a Control diet and Mutants on a HFD. Two-way ANOVA and Tukey’s test. The number of symbols matches the number of zeros in the p value. *P<0.01; **p<0.001; ***p<0.0001; ****p<0.00004 and so on. #=p<0.01, ##=p<0.001; ###=<p.0001; ####=p<0.00001; #####=p<0.000001.

Control and somatotrope LEPR-null mutant females gained more weight on the HFD than their counterparts on the CD. **Table 1** also shows that somatotrope LEPR-null mutants on the CD weighed more than the controls on the CD, as previously described (15). However, the mutant females seemed to be protected from excessive weight gain, accumulating less weight than the control females in response to the HFD (**Table 1**, **Figure 1A**). Note that **Figure 1** graphs the changes in weight over time, and thus the starting number on Week 0 is 0. Although significant weight gain was observed in control females after 2 weeks on the HFD, weight gain in somatotrope LEPR-null female mutants did not become significant until week 3. The mutant females continued to gain less weight than control females from the HFD over weeks 5-16 (**Figure 1A**) and ultimately gained an average of only 16 g (**Table 1**).

**Table 1:** Comparison of weight changes in experimental groups.

Sex and genotype	Experimental group		
Control females	Control diet 16 wks	HFD 16 wks	Significance
Starting weight	27 ± 1.27 (5)	27 ± 1 (5)	NS
Final Weight	33.6 ± 3	50 ± 1.6	Adj P=0.0001
Weight change	6.7 ± 1.9	22.9 ± 2	Adj P=0.0001
Mutant Females	Control Diet 16 wks	HFD 16 wks	Significance
Starting weight	30.3 ± 1.3 (9) *	29.5 ± 0.4 (7) *	NS
Final weight	34.3.5 ± 1.4	47.18 ± 1.98	Adj P<0.0001
Weight change	3.467 ± 0.665	16.64 ± 1.3	Adj P<0.0001

*=Significantly different from control starting weight; Welch's t test-p=0.03.

Weight in grams; number of animals in parentheses.

Starting and final weights (g) after 16 weeks on a HFD. A comparison of control females and mutant females lacking LEPR in somatotropes.

Glucose Tolerance Testing (GTT) indicated that control females on the HFD experienced a significantly greater rise in blood glucose and a delayed reduction over time compared to those on a control diet (**Figure 1B**). In contrast, mutant females on a CD exhibited a slightly higher rise in glucose than the controls, although the reduction over time was similar to that of the controls. The initial increase in glucose among mutants on a HFD was comparable to that of mutants on a CD. However, a delayed reduction was observed at 90 and 120 minutes.

### 3.2 Comprehensive lab animal monitoring

**Figure 2:**
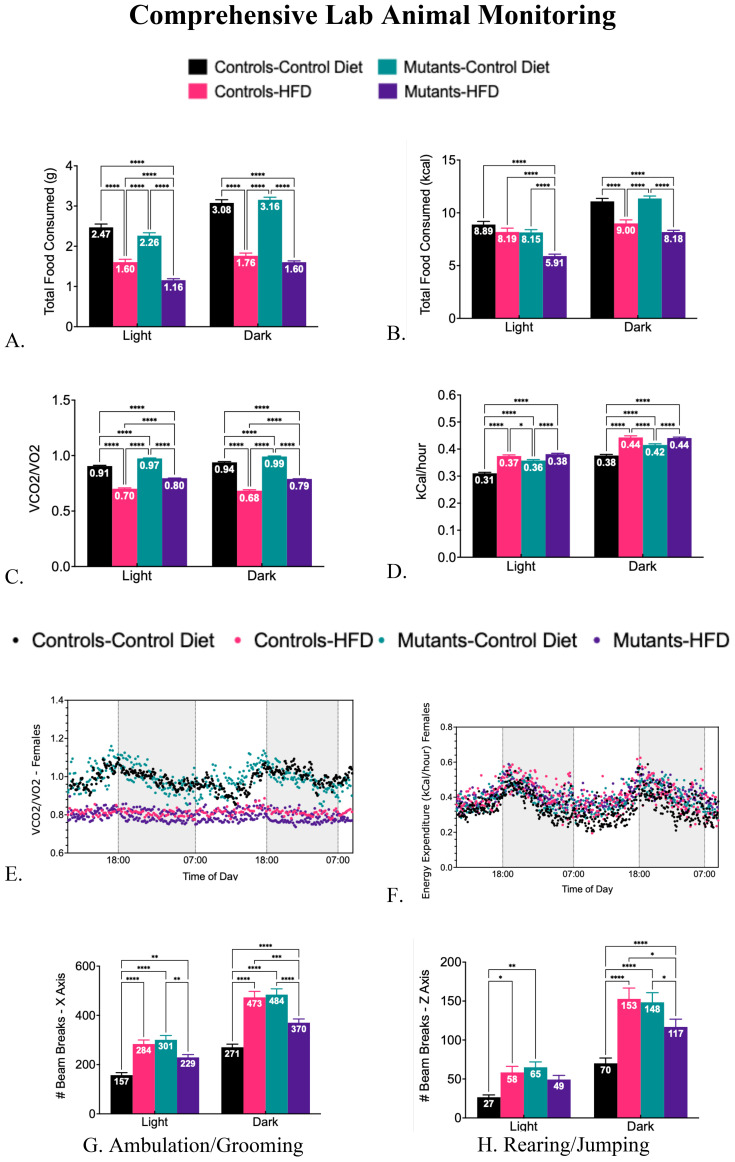
Comprehensive lab animal monitoring tests comparing controls and somatotrope LEPR-null mutants on a control or high-fat diet for 16 weeks. **(A)** compares the total food consumed by weight during the light and dark phases. **(B)** compares the total caloric content of food consumed during the light and dark phases. **(C)** Following a high-fat diet, the reduction in respiratory quotient (VCo2/VO2) indicates preferential fat burning. **(D)** shows energy expenditure in controls and mutants on control or HFD in the light or dark phases. **(E)** shows the Respiratory Quotient over time, comparing mutants and controls after the HFD. **(F)** Energy expenditure of different groups over time. **(G)** Movement in the X-Axis (ambulation and grooming) comparing controls and mutants after a control diet or HFD in the light or dark phases. **(H)** Movement in the Y axis (rearing and jumping) comparing controls or mutants after a control diet or HFD in light or dark phases. Significance indicated by stars after Two-way ANOVA and Bonferroni’s test. The number of symbols matches the number of zeros in the p value. *P<0.01; **p<0.001; ***p<0.0001; ****p<0.00004 and so on.

Metabolic health was monitored in CLAMS cages (Oxymax, Columbus Instruments) (14, 29). Both control and mutant females consumed less food on the HFD, as evaluated by weight (**Figure 2A**) or caloric content (**Figure 2B**). Mutant females ingested less food than controls. The Respiratory Quotient (RQ), measured by the CLAMS unit (ratio of VCO2/VO2), is low in both control and mutant groups on the HFD, indicating increased preference for fat burning as the primary fuel source instead of carbohydrates (**Figure 2C**). As reported previously (14, 15), mutant females on a control diet exhibited slightly higher RQ values than controls on a control diet during both light and dark phases (**Figure 2C**), potentially reflecting an increased use of carbohydrates as fuel in mutants due to the GH deficiency (GH is an important lipolytic hormone). **Figure 2E** illustrates the changes in RQ over time. Both controls and mutants displayed slight changes in energy expenditure with the HFD (**Figure 2D**). **Figure 2F** graphs this over time.

Within the CLAMS cages, activity is measured by detecting the number of light beam breaks in the horizontal plane (walking or grooming- X axis) or vertical plane (rearing or jumping- Z axis). As expected, the mice are more active at night than during the light phase. Overall, the activity pattern has notable genotype differences (the number of beam breaks is represented in the bar) (**Figures 2G, H**). Control females on the HFD exhibited higher activity compared to control females on the control diet during both light and dark phases, while mutants on a control diet showed activity levels like those of controls on an HFD across all phases. Mutants on a HFD demonstrated reduced ambulation and rearing or jumping during the dark phase, although they were more active than controls on a control diet.

### 3.3 Serum levels of cytokines and adipokines

**Figure 3:**
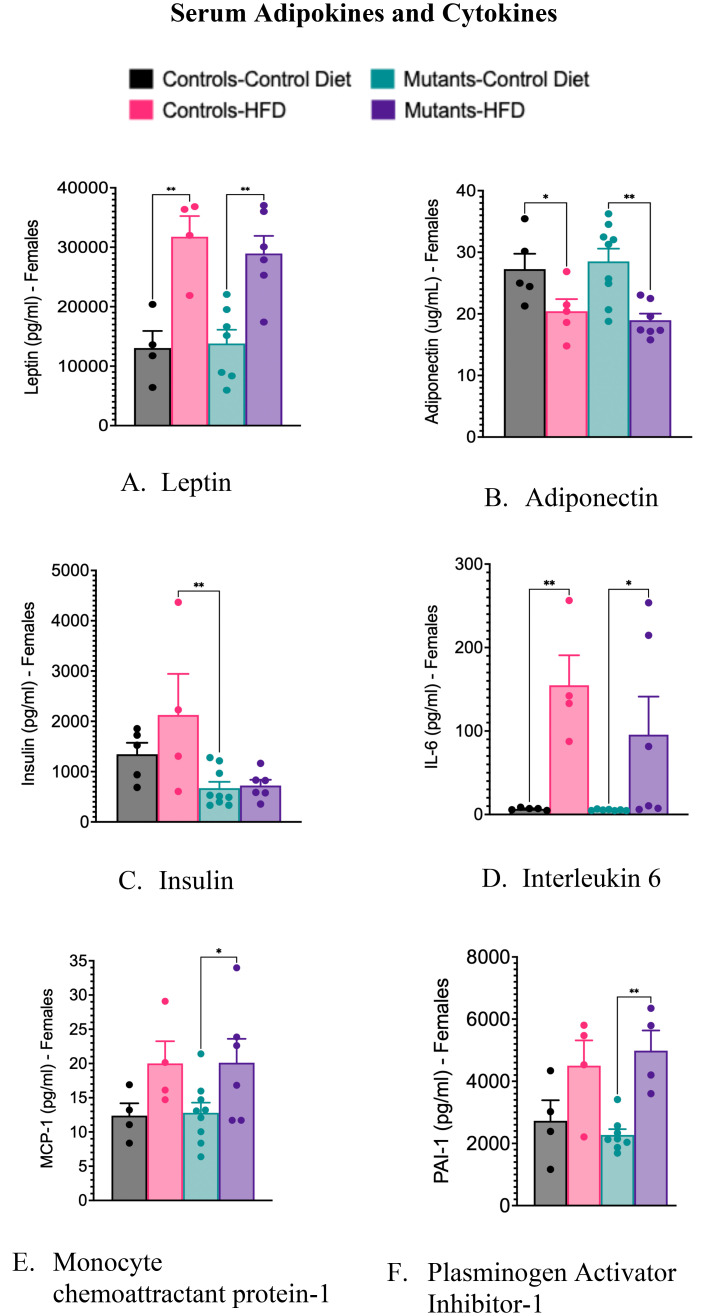
Serum levels of adipokines and cytokines, comparing controls and somatotrope LEPR-null mutants after control or High-fat diet for 16 weeks. **(A)** Serum Leptin; **(B)** Serum adiponectin; **(C)** Serum insulin; **(D)** Serum interleukin 6. Significance indicated by stars after one-way ANOVA and Fisher's Least Significant Difference Test; **(E)** Monocyte chemoattractant protein-1; **(F)** Plasminogen Activator Inhibitor-1. Significance indicated by stars after Two-way ANOVA and Bonferroni’s test. The number of symbols matches the number of zeros in the p value. *P<0.01; **p<0.001.

Consistent with our previous study in young mice (28), a HFD increased serum leptin levels (**Figure 3A**) and decreased serum adiponectin levels (**Figure 3B**) in these older control and mutant mice. The absence of leptin receptors (LEPR) in somatotropes did not affect serum levels of cytokines, except for insulin, which was significantly reduced in mutants (**Figure 3C**). Controls showed no significant change in insulin levels after the HFD (**Figure 3C**). The lower insulin levels in mutants correlate with reduced weight gain (**Figure 1A**) and improved glucose tolerance (**Figure 1B**).

The HFD caused elevated serum levels of the inflammatory marker IL-6 in both control and mutant females (**Figure 3D**). It also increased serum levels of monocyte chemoattractant protein-1(MCP-1) (**Figure 3E**) and plasminogen activator inhibitor-1 (PAI-1) (**Figure 3F**), but significance was observed only in mutant females on a HFD. Unlike the data previously shown in the younger group of mice (28), these older females did not exhibit significant changes in levels of tumor necrosis factor-alpha (TNFα) or resistin following the HFD (data not shown).

### 3.4 Serum levels of pituitary hormones

**Figure 4:**
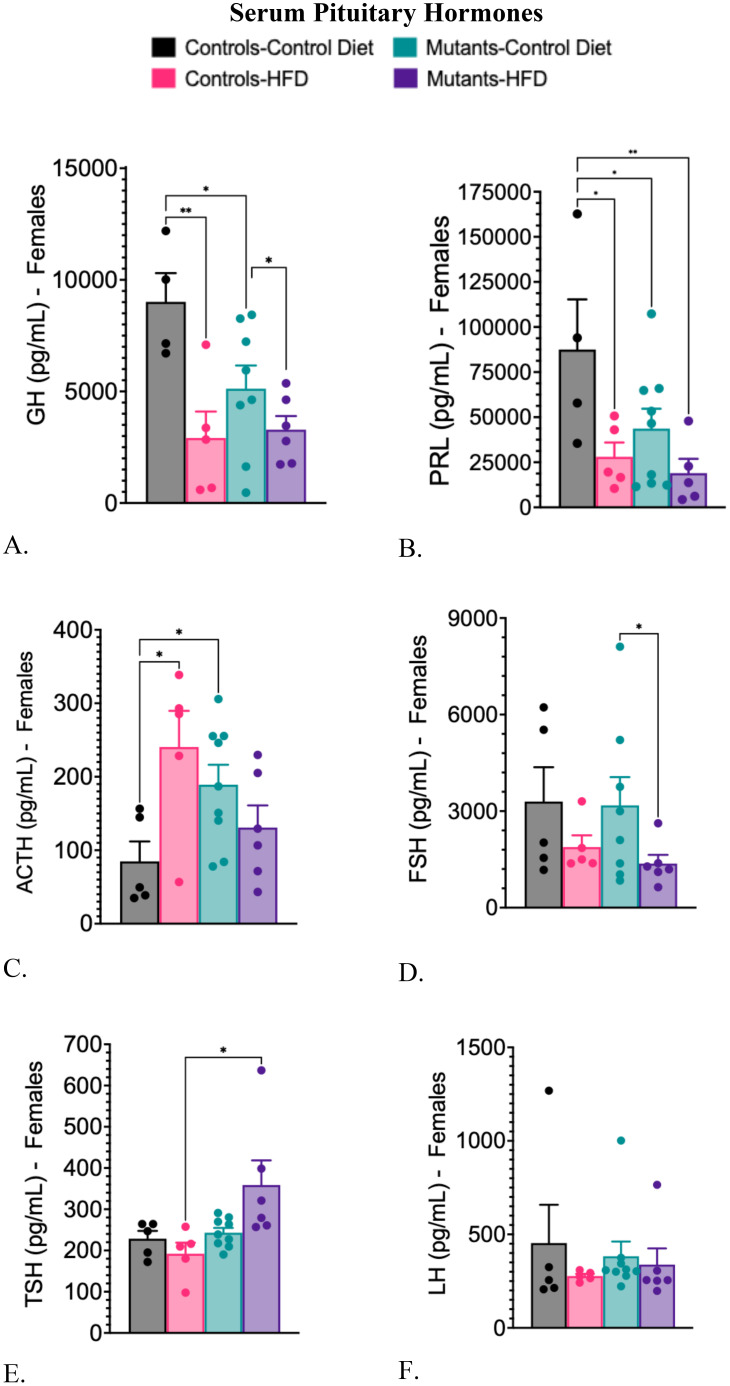
Serum levels of pituitary hormones comparing controls and somatotrope LEPR-null mutants after control or high-fat diet for 16 weeks. **(A)** Serum GH; **(B)** Serum Prolactin; **(C)** Serum ACTH; **(D)** Serum FSH. **(E)** Serum TSH; **(F)** Serum LH Significance indicated by stars after one-way ANOVA and Fisher’s Least Significant Difference Test. The number of symbols matches the number of zeros in the p value. *P<0.01; **p<0.001.

Controls on a HFD exhibited reduced serum GH and PRL levels and elevated serum ACTH levels when compared to controls on a CD (**Figures 4A–C**). Similar results for these three hormones were seen in mutants on a CD (**Figures 4A–C**). The HFD also decreased GH and PRL serum levels in mutants (**Figures 4A, B**). Serum levels of FSH decreased significantly only in mutants on a HFD (**Figure 4D**). Levels of thyroid-stimulating hormone (TSH) rose in mutants on an HFD (**Figure 4E**), compared with controls on a HFD. Serum levels of luteinizing hormone (LH) remained unchanged across all groups (**Figure 4F**).

### 3.5 Mechanistic insights from single-cell transcriptomics

We extrapolated the mechanisms by which aging and loss of leptin signaling may modulate the effect of the HFD on pituitary function from the identified changes in gene expression. As stated in the methods, single-cell RNA sequencing of the pituitary utilized two concurrently processed duplicate pools for each experimental group. Following principal component analysis, the two pools within experimental groups were combined bioinformatically for downstream analysis. We identified and clustered cell populations computationally based on the shared nearest neighbor algorithm using Seurat, as described previously (11, 13, 28). Hormone-producing pituitary cell types, along with pituitary stem and progenitor cells, formed all classical clusters, as shown in the UMAP graph in **Supplementary Figure S1** (57).

### 3.6 Somatotrope clusters

**Figure 5:**
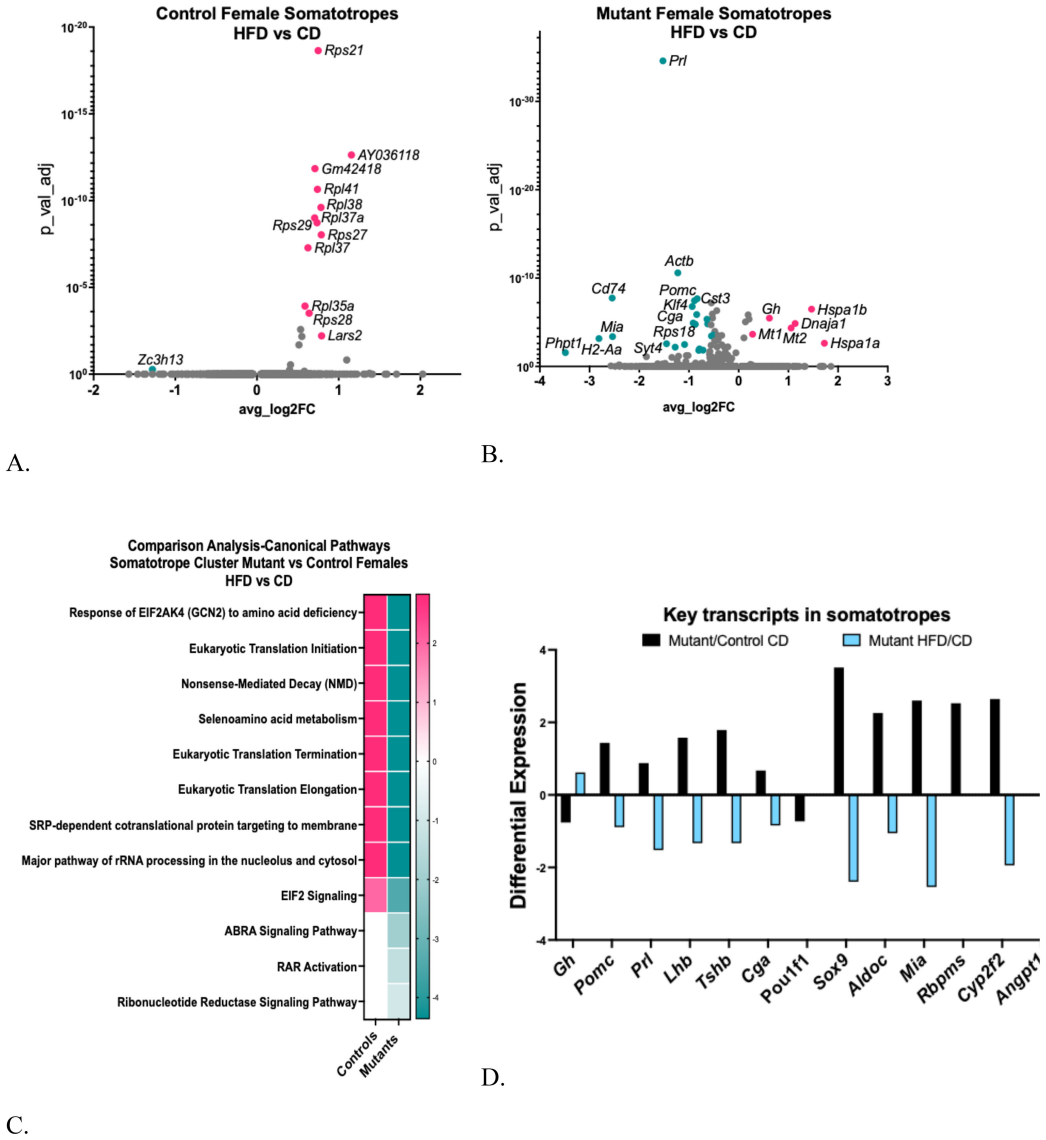
**(A)** Volcano plot showing DEGs in control female somatotrope clusters, comparing HFD/CD. **(B)** Volcano plot showing DEGs in female somatotrope LEPR-null mutant somatotrope cluster comparing HFD/CD. **(C)** Ingenuity Pathway Comparison Analysis of canonical pathways represented by DEGs in somatotrope clusters from somatotrope LEPR-null mutants and control females, comparing HFD/CD for each group. **(D)**. Expression levels of critical pituitary transcripts (pituitary hormones and genes that are markers for progenitor cells) in somatotrope clusters. The graph compares somatotrope LEPR-null mutants after a control diet (black) or a HFD (blue). Expression cutoffs are p<0.055 and Log2FC=0.58, which equals 1.5.

Changes in the control female somatotrope cluster following the HFD were modest and included the upregulation of 12 ribosomal protein genes and a tRNA-synthase [**Figure 5A**, **Supplementary Table S2A** (57)]. However modest, these changes were more numerous than those in the younger female somatotropes (28). In contrast, the mutant female somatotropes were more impacted by the HFD, exhibiting five upregulated DEGs and 45 downregulated DEGs [**Supplementary Tables S2B** (57), **Figure 5B**]. A Gene Ontology analysis of the mutant DEGs indicated that the 45 downregulated DEGs were associated with cytoplasmic translation, signaling, and cell differentiation processes [**Supplementary Table S2C** (57)].

We also analyzed the DEGs using Ingenuity Pathway Analysis (IPA), comparing the canonical pathways represented by DEGs from control and mutant females following a HFD (**Figure 5C**). Translational regulatory pathways are upregulated in control females on a HFD, reflecting the upregulation of ribosomal proteins (**Figure 5A**). In contrast, these regulatory pathways are downregulated in the mutant somatotropes on a HFD, alongside the actin-binding Rho-activating protein pathway, the Retinoic Acid Receptor pathway, and the Ribonucleotide Reductase signaling pathway (**Figure 5C**).

Our recent publication reported that mutant somatotropes, compared to control somatotropes on a control diet, exhibit a gene profile resembling that of progenitor and stem cells (13). This profile included gene markers for progenitor/stem cells as well as genes typically expressed by non-somatotrope cell types, such as *Pomc, Prl, Tshb*, and *Cga* (**Figure 5D**). Notably, somatotropes from mutant females on a control diet exhibited upregulation of Sox9, a key marker for differentiating progenitor cell (7, 8). It is noteworthy that the somatotrope-specific gene markers *Gh* and *Pou1f1* are down-regulated in mutant somatotropes on a control diet, which further supports their immature state (13).

However, mutant somatotropes on a HFD experienced significant changes. **Figure 5D** illustrates that not only did the HFD reverse the differential expression of the progenitor and multihormonal genes, it reduced all but *Pou1f*1 to levels below those observed in the mutants on a control diet. In contrast, *Gh* stands out among the DEGs upregulated by HFD in the mutant, which was unexpected given the reduced serum levels of GH in response to HFD [**Supplementary Table S2B** (57) and **Figure 4B**].

### 3.7 Lactotrope clusters

**Figure 6:**
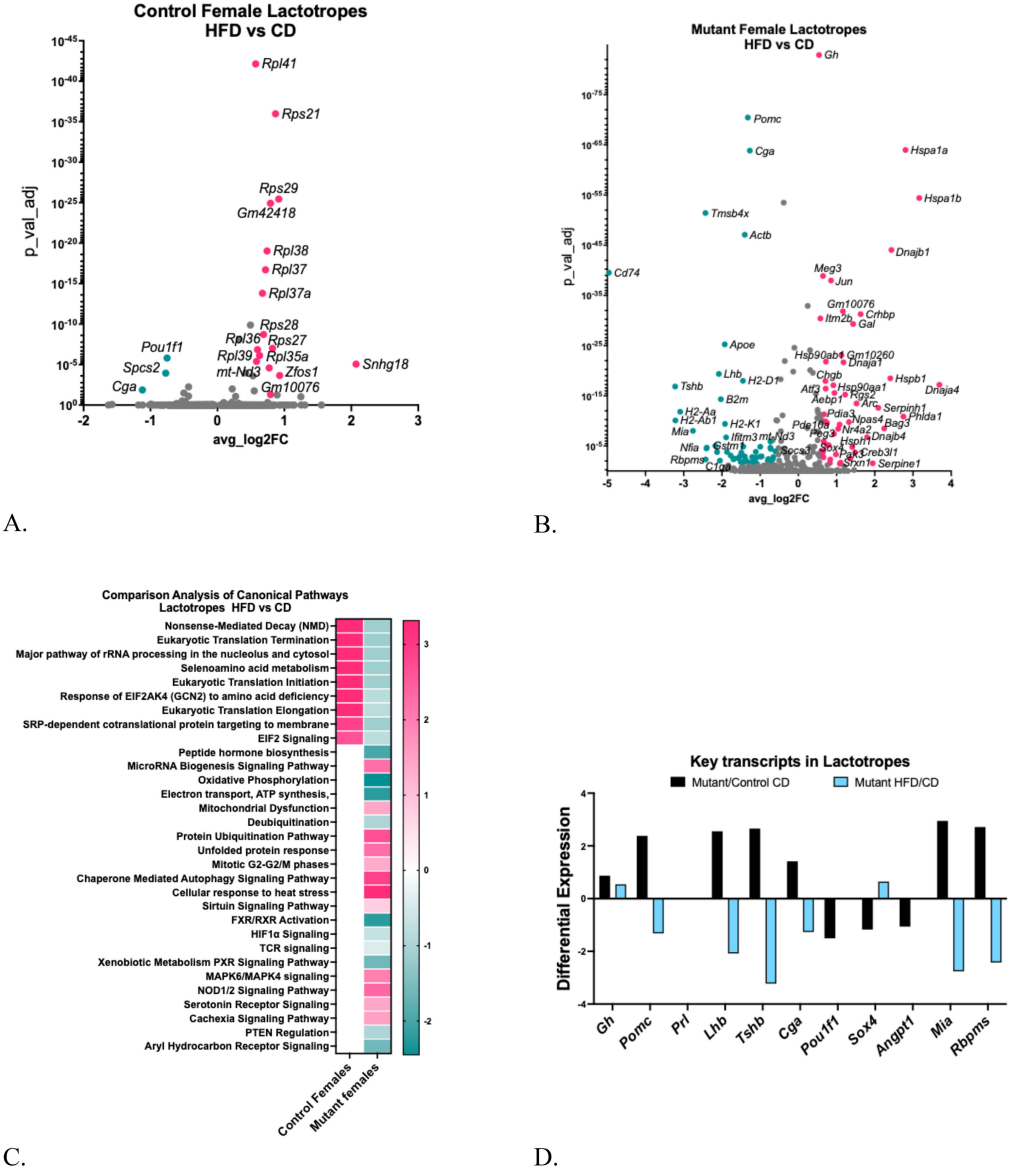
**(A)** Volcano plot showing DEGs in control female lactotrope clusters, comparing HFD/CD. **(B)** Volcano plot showing DEGs in female somatotrope LEPR-null mutant lactotrope cluster comparing HFD/CD. **(C)** Ingenuity Pathway Comparison Analysis of Canonical pathways represented by DEGs in lactotrope clusters from somatotrope LEPR-null mutants and control females, comparing HFD/CD for each group. **(D)** Expression levels of critical pituitary transcripts (pituitary hormones and genes that are markers for progenitor cells) in lactotrope clusters. Graph compares somatotrope LEPR-null mutants after a control diet (black) or a HFD (blue). Expression cutoffs are p<0.055 and Log2FC=0.58, which equals 1.5.

The HFD reduced serum PRL levels in both control and mutant mice after the HFD (**Figure 4B**). **Figure 6A** shows that *Pou1f1* levels are also decreased in the lactotrope cluster from the control females on a HFD, which could contribute to the reduction in serum PRL, as POU1F1 is crucial for the transcription of the *Prl* gene [**Supplementary Table S3A** (57)]. A Gene Ontology Analysis of control mice on a HFD (versus CD) [**Supplementary Table S3B** (57)] revealed that 22 upregulated genes were associated with translational processes and transmembrane transport.

Like somatotropes, the lactotrope cluster from the somatotrope LEPR-null mutants exhibited a stronger response to the HFD compared to the CD [**Supplementary Tables S3C, D** (57)], with over 75 upregulated or downregulated DEGs (**Figure 6B**). The Gene Ontology analysis revealed downregulated genes linked to signaling and cell differentiation, as well as upregulated genes related to protein maturation and folding processes [**Supplementary Tables S3E, F** (57)]. The IPA analysis identified canonical pathways represented by the DEGs in lactotropes, some of which indicate mechanisms underlying the reduced serum prolactin (**Figure 6C**). Following the HFD, control lactotropes showed upregulation in translational pathways, as expected due to the large number of increased genes encoding ribosomal proteins (**Figure 6A**). In contrast, mutant lactotropes after an HFD exhibited significant reductions in translational pathways and pathways crucial for oxidative phosphorylation, peptide hormone biosynthesis, signaling, and secretion. Like lactotropes in young control females (28), HFD-upregulated DEGs included those representing mitochondrial dysfunction. Moreover, mutant female lactotropes demonstrated upregulation of DEGs in pathways indicative of rough endoplasmic reticulum stress, including the unfolded protein response, the cellular response to heat stress, and autophagy (**Figure 6C**).

As reported for somatotropes (13), the lactotrope cluster from mutants also exhibited increased differential expression of multiple pituitary hormone transcripts as well as a group of genes with an immature or progenitor-cell signature (**Figure 6D**). However, Prolactin (*Prl*) was not increased in this population. The other increased pituitary hormone transcripts include *Gh, Pomc, Lhb, Tshb*, and *Cga*, and the immature genes that were increased included *Angpt1*, *Mia*, and *Rpms*.

However, *Pomc*, *Lhb*, *Tshb* and *Cga* genes were significantly reduced in mutants exposed to a HFD (**Figure 6D**). Among progenitor genes, *Sox4* was increased by HFD and *Mia* and *Rbpms* were both reduced. We also investigated changes in these same key transcripts in **Figure 5D** in lactotropes from controls on a HFD and found that *Cga* is reduced by -1.12 (adjusted p=0.01), and *Pou1f1* is reduced by -0.75 (adjusted p=1.55E-06). The reduction in *Pou1f1* correlates well with the decreased serum prolactin observed in the older control females on a HFD.

### 3.8 Corticotrope cluster

A high-fat diet (HFD) increased serum levels of adrenocorticotropic hormone (ACTH) in the older control females (**Figure 4C**). Despite this change, the corticotrope cluster in control females on an HFD showed no differentially expressed genes (DEGs) that met the significance threshold.

**Figure 7:**
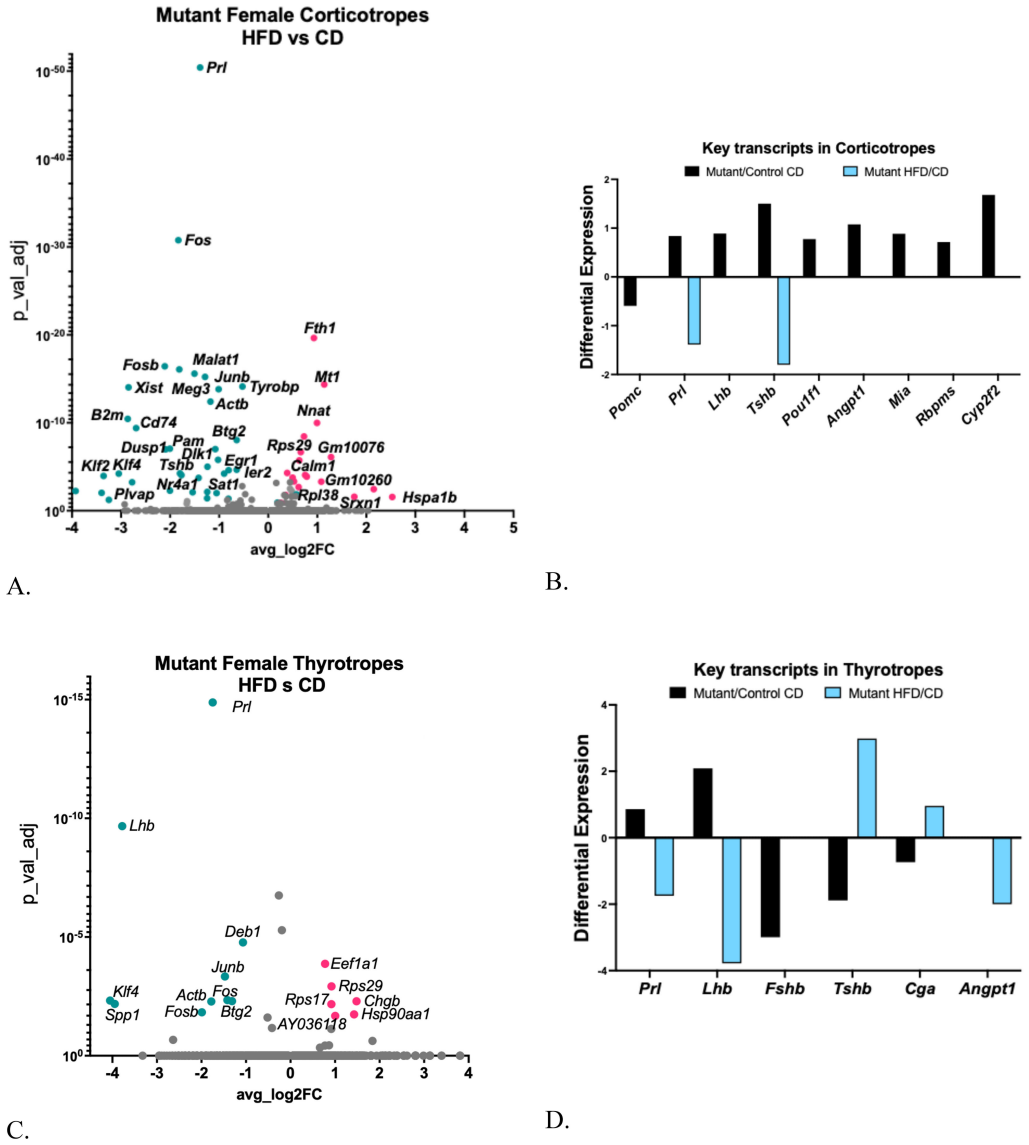
**(A)** Volcano plot showing DEGs in somatotrope LEPR-null mutant corticotrope cluster comparing HFD with CD. **(B)** Expression levels of critical pituitary transcripts (pituitary hormones and genes that are markers for progenitor cells) in corticotrope clusters. Graph compares somatotrope LEPR-null mutants after a control diet (pink) or a HFD (turquoise). Expression cutoffs are p<0.055 and Log2FC=0.58, which equals 1.5 **(C)** Volcano plot showing DEGs in somatotrope LEPR-null mutant thyrotrope cluster comparing HFD with CD. **(D)** Expression levels of critical pituitary transcripts (pituitary hormones and genes that are markers for progenitor cells) in thyrotrope clusters. Graph compares somatotrope LEPR-null mutants after a control diet (black) or a HFD (blue). Expression cutoffs are p<0.055 and Log2FC=0.58, which equals 1.5.

Mutant females on a CD also exhibited higher serum ACTH levels compared to the controls on the CD (**Figure 4C**), which was unexpected because *Pomc* expression is reduced in the mutant (**Figure 7A**). When exposed to a HFD, corticotropes from mutants showed 26 upregulated DEGs and 36 downregulated DEGs [**Supplementary Table S4A** (57), **Figure 7A**], and no differential expression of *Pomc* (**Figure 7B**). Gene Ontology analysis revealed increases in DEGs related to signaling, transcription, and cell differentiation pathways [**Supplementary Table 4C** (57)], along with decreases in DEGs associated with cytoplasmic translation [**Supplementary Table S4D** (57)].

Corticotropes from mutants on a CD also exhibited an increase in a subset of key transcripts, including other pituitary hormones and progenitor cell markers such as *Prl, Lhb, Tshb, Pou1f1*, and four progenitor gene markers (**Figure 7B**). The differential increase in progenitor cell genes, *Lhb* and *Pou1f1*, was no longer evident in corticotropes from mutants on a HFD. However, the HFD further decreased the expression of *Prl* and *Tshb* (**Figures 7A, B**).

### 3.9 Thyrotrope cluster

Serum TSH remained unchanged in controls following the HFD but increased in mutants (**Figure 4E**). In the control females on a HFD, thyrotropes showed a significant decrease in *Gh* (log2FC -1.8; adjusted p=0.00526) and an increase in ribosomal subunit protein 27 (1.27-fold, adjusted p=0.03). In contrast, the mutant females displayed more DEGs in thyrotropes following a HFD [**Figure 7C**; **Supplementary Table S4B** (57)]. However, the DEGs were not sufficiently numerous for an IPA analysis. Notably, like somatotropes, lactotropes, and corticotropes, thyrotropes from mutants on a control diet showed multihormonal expression with upregulation of *Lhb* and *Prl and* downregulation of *Fshb*, *Tshb*, and *Cga* (**Figure 7D**), but no change in the stem cell genes. Some of these changes were reversed in HFD mutants, including downregulated *Prl, Lhb, and Angpt1.* The upregulated *Tshb* and *Cga* correlated with the increased serum TSH (**Figure 4E**) in the mutants.

### 3.10 Gonadotrope cluster

A high-fat diet (HFD) reduced serum FSH levels in mutants, with the values also showing a trend toward reduction in controls. The HFD in controls showed increased *Lhb* and decreased *Fshb*, *Prl, Nnat, and Rbp4* [**Supplementary Table S5** (57)]. Despite the reduced serum FSH, gonadotropes in mutants on an HFD exhibited no differentially expressed genes (DEGs) that met the significance threshold.

### 3.11 Sox2 stem cell cluster

**Figure 8:**
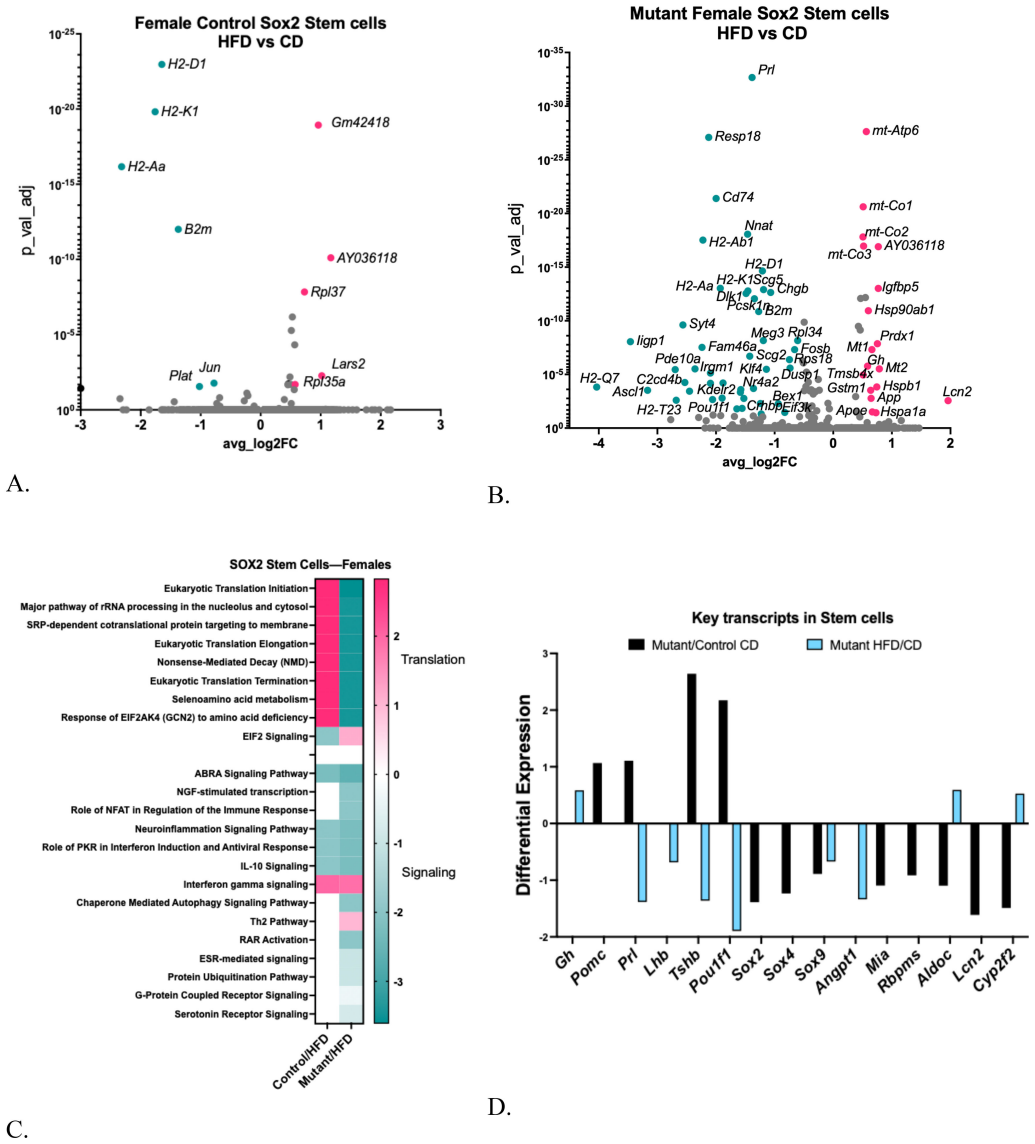
**(A)** Volcano plot showing DEGs in control female Sox2 stem cell clusters comparing HFD with CD. **(B)** Volcano plot showing DEGs in somatotrope LEPR-null mutant Sox2 Stem cell cluster comparing HFD with CD. **(C)** Ingenuity Pathway Comparison Analysis of Canonical pathways represented by DEGs in Sox2 stem cell cluster in somatotrope LEPR-null mutants or control females, comparing HFD/CD for each group. **(D)** Expression levels of critical pituitary transcripts (pituitary hormones and genes that are markers for progenitor cells) in Sox2 Stem cell clusters. Graph compares somatotrope LEPR-null mutants after a control diet (black) or a HFD (blue). Expression cutoffs are p<0.055 and Log2FC=0.58, which equals 1.5.

Previously, we reported significant changes in DEGs within the *Sox2*-positive stem cell cluster of young mice exposed to a high-fat diet (HFD) (28), supporting studies from others that indicate stem cells are susceptible to inflammatory and oxidative stress (10). However, in the older group of control females, a HFD caused a modest change in DEGs [**Figure 8A**, **Supplementary Table S6A** (57)].

In contrast, like other mutant female clusters on a HFD, the *Sox2* stem cells exhibited a greater number of differentially expressed genes (DEGs). Most of these genes were downregulated compared to the mutants on the control diet [**Figure 8B**, **Supplementary Table S6B** (57)]. A Gene Ontology analysis revealed significant downregulation of DEGs related to signaling, cell differentiation, and immune or defense responses [**Supplementary Table S6C** (57)]. The IPA analysis of DEGs in *Sox2* stem cells indicated significant increases in translation pathways in control females following a HFD. In contrast, numerous DEGs in stem cells from mutants on a HFD are downregulated, including those in translational regulatory pathways and several signaling pathways (**Figure 8C**).

**Figure 8D** illustrates the same group of key transcripts examined in the hormone-bearing clusters. Note that Sox2 stem cells from mutants on a control diet exhibited increases in *Pomc, Prl, Tshb*, and *Pou1f1*, indicating some differentiation within the stem cell population. This is consistent with activation of the stem cells, although the major known activator, IL-6 (4, 10, 34–36, 58), was not elevated in the mutant Sox2 cells. Along with the increases in pituitary hormone genes, there are decreases in progenitor cell markers, including *Sox2, Sox4, Sox9, Mia, Rbpms, Aldoc, Lcn2, and Cpt2f2* in the mutant stem cells (**Figure 8D**), which is also consistent with activation. Exposing the mutants to the HFD reversed some of these changes in Sox2 stem cells, including *Prl, Tshb and Pou1f1.* In contrast, the HFD increased *Gh*, and slightly increased *Rbpms, Aldoc*, and *Cyp2f2* (**Figure 8D**). The increase in *Gh* is also consistent with activation (58), which could have been caused by the high serum IL-6 in the mutant on a HFD.

### 3.12 Upstream regulators impacting DEGs in mutants after the HFD

**Figure 9:**
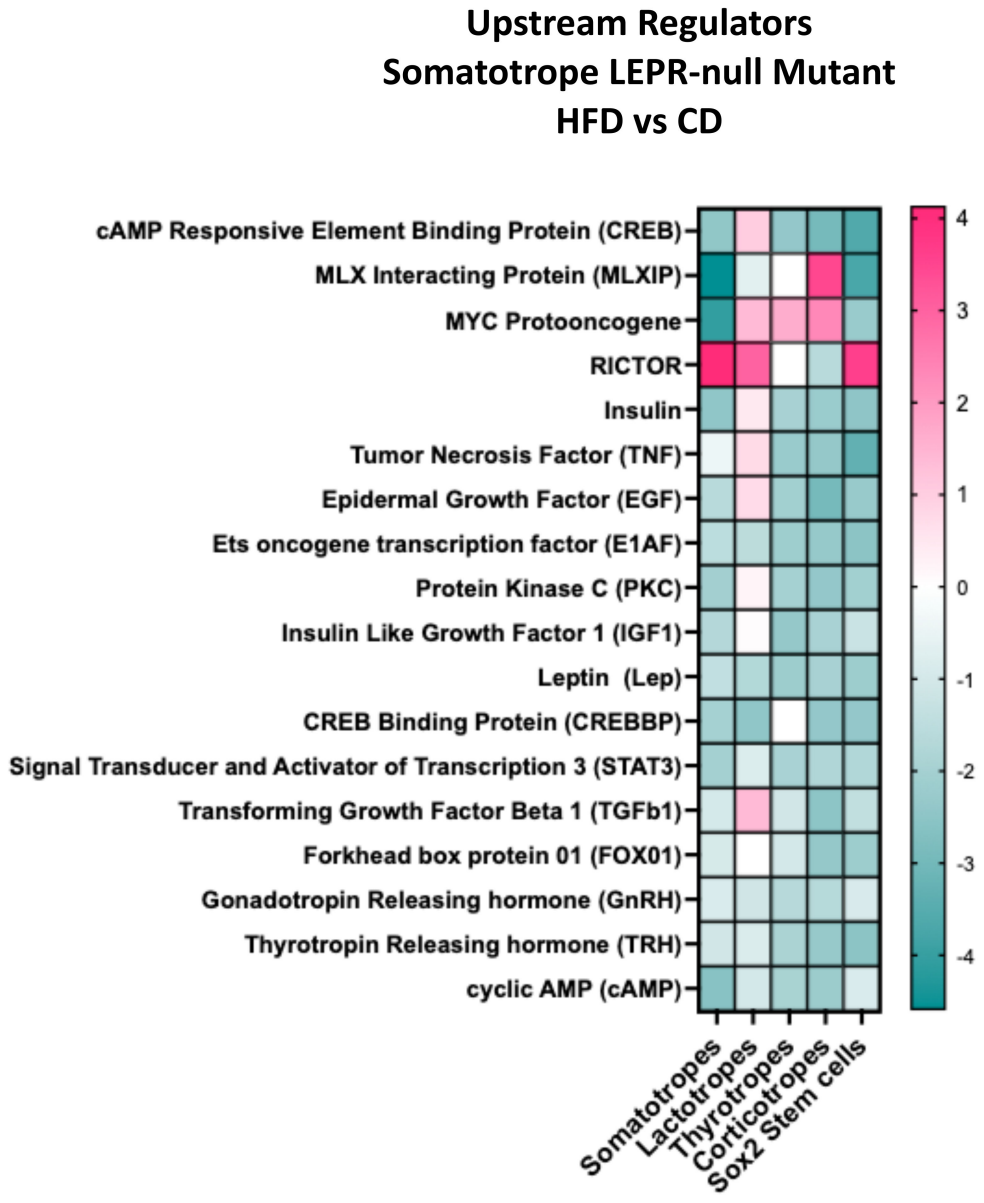
IPA comparison analysis predicted upstream regulators for each cluster based on DEGs. The graph compares the response to HFD/CD in the somatotrope LEPR-null mutants. The Y axis shows the z-score that predicts the direction of the regulation based on the expression of individual target DEGs. See **Supplementary Tables S7**-**S24** (57) for a complete list of the target DEGs from each cluster.

The DEG dataset from each cluster can identify potential upstream regulators predicted to be active in each cell type. **Figure 9** displays the top upstream regulators in clusters from mutants on a HFD. The heatmap z-scores suggest the direction of the regulator's action based on the net change in the target DEGs within that cluster (59). **Supplementary Tables S7**–**S24** (57) list each regulator's target genes, demonstrating differential expression of that gene in each cell type. The heatmap in **Figure 9** shows that most of the regulators have a negative z-score, indicating that the target DEGs are downregulated. Among the DEGs are pituitary hormones, which are highlighted in these tables (57).

We studied this group of target genes to determine if there was differential expression in IL-6 pathway genes that would point to activation of stem cells (10, 34, 35). Il-6 and IL-11 were listed as upstream regulators, but their z scores were negative in the Sox2 stem cells. Furthermore, JAK/STAT pathway regulators Leptin and Stat3 were listed in **Supplementary Tables S17**, **S19**, and their DEG targets were mostly downregulated. Thus, the data does not support activation by these cytokines.

**Figure 10:**
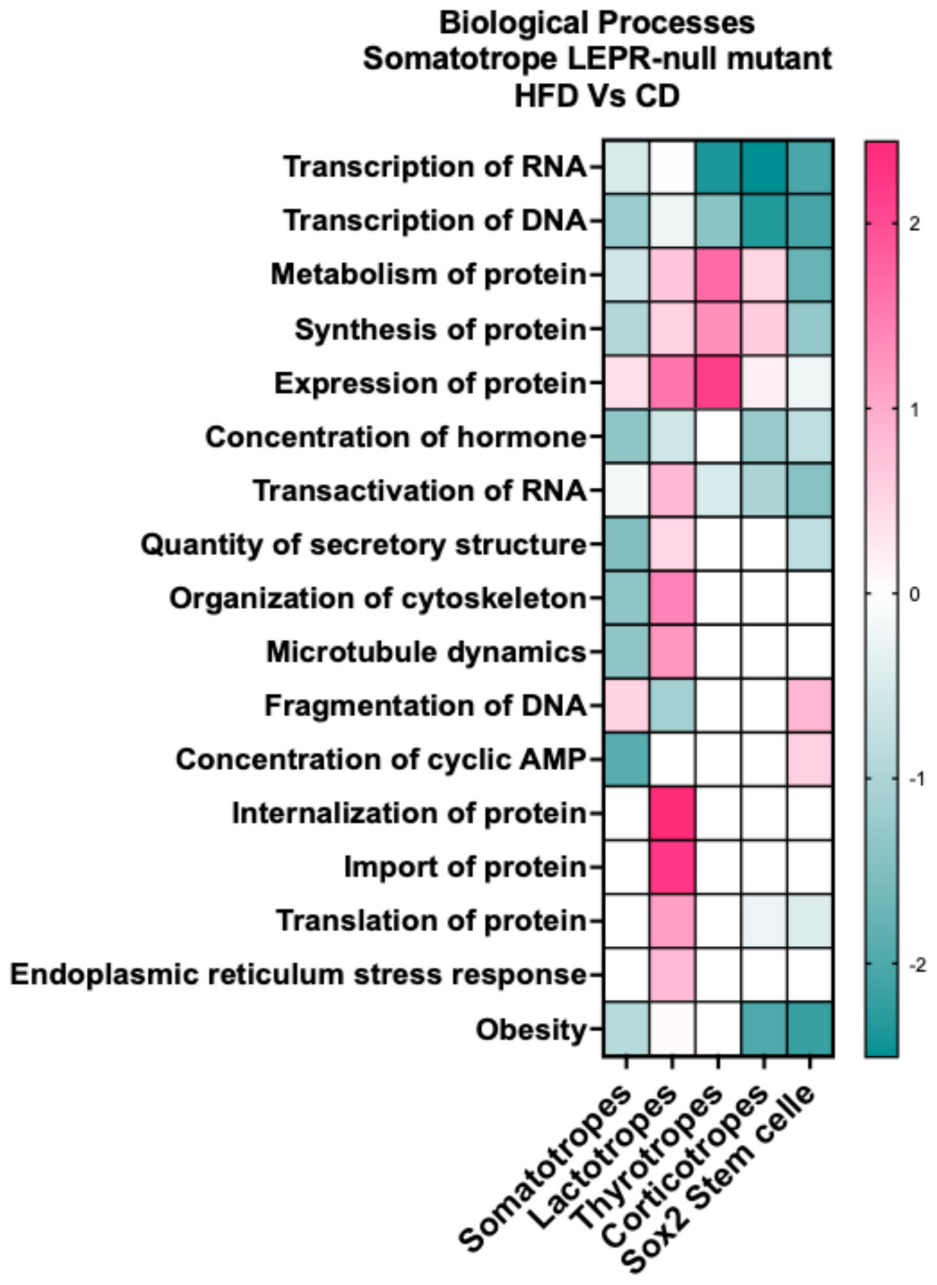
IPA comparison analysis of biological processes indicated from the DEGs in each cluster. The DEGs are in clusters from somatotrope LEPR-null mutants exposed to a HFD (HFD/CD).

There were enough DEGs in somatotrope, lactotrope, thyrotrope, corticotrope, and stem cell clusters from the mutants on a HFD to allow us to predict changes in biological processes within those clusters and compare the responses of each. **Figure 10** illustrates a heatmap indicating that the direction of change in the process may vary with cell type. All clusters, except lactotropes, exhibit DEGs suggesting reduced transcription of RNA and DNA, as well as a decreased concentration of the hormone. Somatotropes and Sox2 stem cells show DEGs indicating diminished protein synthesis and transport. Lactotropes demonstrate increased protein synthesis and transport, along with heightened endoplasmic reticulum stress response. Corticotropes and thyrotropes exhibit a mixture of responses, including increases in metabolism and protein synthesis. Three cell types- somatotropes, corticotropes, and stem cells- are targets of downregulated genes that serve as markers for changes associated with obesity.

## 4 Discussion

### 4.1 Impact of aging vs HFD on somatotrope function

This study focused on the impact of a 16-week HFD feeding on older female mice (10 months old at the time of sacrifice). It follows a recent study of the effects of a HFD on young females, which showed differentially expressed genes (DEGs) primarily in the pituitary lactotrope and stem cell populations (28). Our findings suggest that the older female pituitary cells clusters may be more vulnerable, as the HFD significantly affected more transcripts across most cell clusters.

The GH/IGF-1 axis is vital to the optimization of body composition, maintaining strong bones and muscle and preserving lean mass (60–64). GH is also lipolytic, reducing fat stores and lowering serum leptin levels (60–64). However, GH secretion declines with age by 14% per decade in humans (65). In the control mice in this study, aging to 10 months did not significantly impact the average serum levels of GH. The serum levels of GH in these 10-month-old control females (**Figure 4**) were in the same range as levels in the 5–6 month old mice (28). However, studies by Huang et al. (66) have reported that there is a decline in pulsatile GH from 3 months of age to 4 months of age in control C57Bl/6J male mice. Indeed, the slightly older (4-month) adult mice in their study exhibited heightened vulnerability to a HFD after only 4 weeks, with a significant reduction in total, pulsatile, and basal GH secretion. Thus, it is not surprising that 10-month-old control females in the present study responded to the HFD with lowered serum GH levels.

In addition, other intervening factors that may affect the vulnerability of the 10-month-old control females may be the reduced clustering of somatotropes seen at 16 weeks (4 months) by Bonnefont et al. (67). This is compounded by the reduction in the number of GHRH neurons by 4 months of age in mice (68). The biological implications of these findings is that the aging somatotropes become less resilient to stress, including the oxidative stress of obesity. Also, they are less likely to be regenerated by the stem cell pool, which is not activated *in vivo* in 8 month old mice (34–36, 58).

Our findings, discussed below, will point to dysfunction in the underlying secretory mechanisms within each cell type, which is confirmed by the analysis of DEGs, canonical pathways, and upstream regulators. One of the potential regulators that increases following the HFD is IL-6, a cytokine produced in response to inflammation associated with obesity and a HFD. IL-6 levels were high in this group of mice and in our previous study on young mice (28). Interestingly, IL-6 is known to stimulate PRL, GH, and LH release from normal anterior pituitary cells (69); however, the reduced serum levels of GH and PRL suggest that the cells from our HFD mice were unable to respond to IL-6 modulation. Similarly, leptin has been reported to be trophic for somatotropes, maintaining cell numbers and stores of GH (15, 16). However, the low serum GH in the HFD-exposed control mice suggests that, either the somatotropes are unable to respond to the high leptin because of dysfunctional secretory mechanisms and/or they have become leptin resistant. Future studies of partial leptin reduction (70, 71) may provide more information about the impact of hyperleptinemia on somatotrope function. We sought evidence for upregulated pathways that inhibit leptin action (72, 73) and find that the leptin-inhibitory factor, *Socs3, i*s not upregulated significantly in any of the clusters.

IL-6 is also a potent regulator, which activates stem cells to begin a differentiation process (4, 5, 10, 34, 35, 58) and in young mice, endogenous IL-6 is upregulated in the stem cells before activation (58). However, IL-6 is not upregulated in any of these clusters in the somatotrope Lepr-null pituitary or in response to the HFD, perhaps because the mice are middle-aged. Indeed, Vennekens et al. reported that endogenous IL-6 does not activate stem cells in mice over 8 months of age (58).

The impact of the selective ablation of LEPR in somatotropes has been studied in female mice both at 6 months of age (15, 29) and in younger cohorts (14, 31). Aging tended to exacerbate adult-onset obesity in these female mice (14, 31). Our previous studies reported that somatotrope LEPR-null mutants on a control diet exhibited increased abdominal fat, reduced somatotrope function, and displayed metabolic dysfunction as assessed by indirect calorimetry in metabolic cages (14, 15, 29, 31, 32). This cohort of mice was again evaluated in metabolic cages in the present study, and they showed similar responses to those published previously (14, 29). However, they appeared to be somewhat resistant to the HFD despite their GH deficiency, which will be discussed below.

### 4.2 Reduced weight gain in somatotrope LEPR-null mutants

Our studies of both older and younger (28) control females revealed a similar response to a HFD, including a 20 g weight gain along with reduced serum growth hormone (GH) and prolactin (PRL). In contrast, the mutant females gained less weight (16.64 g) on the HFD, reaching a plateau by 13 weeks. Their lower weight gain correlated with a steeper decline in glucose during the glucose tolerance test (GTT), which was accompanied by lower serum levels of insulin. This reduced weight gain also correlated with reduced food intake and overall higher activity. We have no explanation for the higher serum levels of thyroid-stimulating hormone (TSH) in the mutant females on the HFD (compared with control females on the same diet); further tests of thyroid function are needed.

As previously reported, the mutant mice on a control diet preferentially burn more carbohydrates than fat as assessed via indirect calorimetry in a CLAMS metabolic cage and we have postulated that this is due to their GH deficiency, resulting in significantly lower serum GH, and numbers of cells storing GH proteins (14, 29). GH is lipolytic and reduced GH compromises fat burning. However, despite the reduced serum GH after a HFD, the fat content of the diet itself promoted the preferential burning of fat as fuel in the mutants on a HFD.

Finally, it is interesting that despite the lower levels of diet-induced obesity the pituitary cells from these older mutant females were affected more dramatically at the transcriptional level by the diet and/or obese state than those from control groups. This will be discussed in more detail in the following sections.

### 4.3 Differential gene expression in individual pituitary clusters

#### 4.3.1 Somatotropes

In control females on a HFD, there were too few DEGs to provide insight into the underlying mechanisms for the reduced serum GH. Above, we suggest that the aging-induced reduction in cellular clustering/networking (67) and numbers of GHRH neurons may be contributing factors (68). Ruggiero-Ruff et al. (74) reported the impact of a 12-week HFD on somatotropes from very young C57Bl6 males, which were placed on the HFD at weaning. Their scRNA-seq analysis of somatotropes showed increased numbers of somatotropes and they observed increased expression of *Gh* mRNA-bearing cells (by RNAscope). The pituitary content of *Gh* mRNA and protein were unchanged. These findings may show important age and sex differences in responses to a HFD. It is possible that the younger population may not be subject to hyperleptinemia and may be stimulated by the high leptin (15) and high IL-6 (69). Unexpectedly, we also found an increase in *Gh* mRNA in the somatotrope, lactotrope, and stem cell clusters from mutants on a HFD. The IL-6 levels may have stimulated the increased transcript expression as recent reports show that IL-6 stimulates *Gh* mRNA synthesis in GH3 cells (75).

Mutants lacking LEPR in somatotropes exhibited changes in DEGs, pathways, and processes that may explain the HFD-induced reduction in serum GH. A comparative analysis of canonical pathways in mutant somatotropes revealed that all translational pathways were downregulated by a HFD, suggesting suppressed protein synthesis in the absence of leptin signals. Furthermore, our study of upstream regulators, as illustrated in **Figure 9**, shows negative z-scores, which are derived from the downregulated expression of DEGs, and may suggest that the pathways themselves are downregulated. A review of the literature shows that the following upstream regulators are important for GH secretion: FOX01 (76, 77), cAMP (78), STAT3 (11), CREB (79), and Leptin (11, 14–16, 29). Based on their DEG targets in **Supplementary Tables S7**–**S24** (57), all of these regulatory pathways are predicted to be downregulated in the somatotrope population. Additionally, an examination of the biological processes represented by the DEGs in somatotropes (**Figure 10**) indicated deficiencies in transcription, protein synthesis, concentration, secretory mechanisms, and metabolism.

The loss of LEPR in somatotropes correlates with the down-regulation of a number of leptin signaling pathways and the DEGs in these pathways are among those listed in **Supplementary Tables S7**–**S24** (57). Leptin signaling through the JAK/STAT pathway phosphorylates STAT3, and **Supplementary Table S17** lists the direct targets of leptin, many of which are impacted by the HFD. Importantly, STAT3 targets are listed in **Supplementary Table S19** (57), showing that most are down-regulated, especially in somatotropes. However, an important inhibitor in the leptin signaling pathway, *Socs3*, is not upregulated in LEPR-null somatotropes and is downregulated only in lactotropes and corticotropes from these mutant mice. This evidence suggests that leptin resistance from the hyperleptinemia following a HFD may not be the mechanism behind the reduced serum GH.

#### 4.3.2 Lactotrope clusters

Reduced serum PRL may correlate with elevated free fatty acids from the HFD (80) in both controls and mutants on an HFD. Additionally, we have reported that the lactotropes are particularly sensitive to oxidative stress in the younger female group on a HFD (28). As in the case of the younger cohort (28), the older female lactotropes also show downregulated DEGs indicating reduced oxidative phosphorylation and electron transport pathways, and upregulated DEGs indicating mitochondrial dysfunction.

The lower serum prolactin in control females on a HFD can be correlated with the reduced *Pou1f1* in the lactotrope cluster, which is vital for *Prl* transcription. However, *Prl* is not reduced in this cluster. Low serum prolactin also correlates with the downregulation of the peptide hormone biosynthesis canonical pathway and the downregulation of upstream regulators known to affect lactotropes, including leptin and STAT3 (81), TRH (82), cAMP (83), and CREBBP (84). DEGs associated with DNA fragmentation and metabolism are also downregulated in lactotropes. RNA-scope studies of young male mouse lactotropes by Ruggiero-Ruff et al. (74) showed that there were no changes in percentages of lactotropes (by RNA-scope but reduced pituitary content of Prl and mRNA following the HFD.

Whereas the *Prl* gene is not reduced in the prolactin cluster from these older females, evidence exists for the downregulation of the *Prl* gene as observed in several non-lactotrope clusters from the mutant females on a HFD, including somatotropes, corticotropes, thyrotropes, and Sox2 stem cells. This evidence suggests that the HFD may suppress the expression of multihormonal genes in the progenitor cells of these clusters, potentially preventing the production of prolactin, which would normalize serum prolactin levels.

#### 4.3.3 Corticotropes

In young females on a high-fat diet (HFD), corticotropes exhibited no differentially expressed genes (DEGs), and serum levels of adrenocorticotropic hormone (ACTH) were normal (28). In young males on a HFD Ruggiero-Ruff et al. reported that important genes related to nuclear receptors *Nr4a1* and *Nr4a2* were downregulated in corticotropes along with other DEGs (74).

The corticotropes in older control females may be more susceptible to oxidative stress of obesity, which could account for the elevated serum ACTH levels. A notable upstream regulator for DEGs in mutant female corticotropes is the Myc proto-oncogene (Myc), which promotes cell growth and differentiation. This finding aligns with findings from a study of 33 human pituitary tumors, indicating that an ACTH-secreting tumor specifically expressed Myc (85). The upregulated target DEGs also include numerous ribosomal proteins, reflecting increased protein metabolism and synthesis in the corticotrope cluster. Collectively, these factors may explain the heightened serum ACTH levels in the mutants.

#### 4.3.4 Thyrotropes

Thyrotropes from control females also had no DEGs following a HFD in young females (28), and in the present study of control older females on a HFD. In young males from the study by Ruggiero-Ruff et al, there was a modest number of DEGs; however, none were involved in TSH transcriptional regulation (74). Thyrotropes from mutant females on a HFD had a modest number of DEGs that predicted reduced responses to the upstream regulators shown in **Figure 9**, including TRH. Notably, the reductions in transcription factors *Junb*, *Fos*, and *Fosb* support these reduced responses (86). Thyrotropes from mutant females on a HFD also exhibited reductions in DEGs related to RNA transcription and DNA and RNA transactivation despite the high serum TSH levels following a HFD.

#### 4.3.5 Gonadotropes

Although serum FSH was reduced in the mutants on a HFD, the gonadotropes showed the fewest DEGs following the HFD compared to all pituitary cell types, as reported previously with younger females on HFD (28). Due to the limited number of DEGs, it was not possible to identify pathways and upstream regulators that resulted in the decreased serum FSH.

Interestingly, the scRNA-seq study by Ruggiero-Ruff et al. of young males (74) showed significant numbers of DEGs in the gonadotrope cluster, with downregulation of *Lhb, Gnrhr*, as well as important transcriptional factors, including *Fos, Jun, Egr1*, and *Atf3* (74). No changes were seen in *Fshb, Foxl2* and *Nr5a1.* These findings highlight important sex and age differences in the gonadotrope response to a HFD.

#### 4.3.6 Sox2-positive Stem cells

Pituitary *Sox2*-positive stem cells in this study of older control females exhibited fewer DEGs following a HFD compared to young females (28), In mutant females, the HFD decreased translational pathways, as well as those that support the inflammatory response, estrogen receptor signaling, and G protein-coupled receptor signaling. Although the mutant female stem cells appeared to show signs of increased differentiation on a control diet, this effect is partially reversed in mutants exposed to the HFD. Previous studies have shown that Sox2 stem cell activation may be caused by expression of IL-6 and activation of the JAK/STAT3 pathway (58). However, as expected from the age of these mice (34, 36, 58) there is no evidence for elevated Il-6 transcripts. The elevated serum levels of IL-6 in the mutant on a HFD could have stimulated the expression of *Gh* in this population, however. One might speculate that the stem cells are being adaptively differentiated to produce somatotropes, in response to the deficiency both in the mutant and mutant on a HFD (4, 7, 8). This differentiation might also involve blocking other pituitary hormones, which would explain the reduction in *Prl*, *Tshb*, and *Lhb* in this population.

### 4.4 The impact of ablation of LEPR in somatotropes on pituitary plasticity

As reported above and previously (13), the multihormonal and progenitor cell DEGs in the mutant mice suggest that some somatotropes in that population were immature. This hypothesis is based on the significant increase in the expression of non-somatotrope hormone genes, including *Pomc, Lhb, Prl, and Cga*, along with an increase in the gene encoding the progenitor/stem cell marker, Sox9. *Sox9* is normally expressed later than *Sox2* during development in a population of progenitor cells in the embryo, neonate, and adult pituitary (7, 8, 87, 88). Further evidence for immaturity was found by the upregulation of genes that are usually restricted to or most highly expressed in stem cells, including those encoding Melanoma Inhibitory Activity (*Mia)*, RNA-binding protein with multiple splicing (*Rbpms*), Aldolase Fructose Biophosphate C (*Aldoc)*, and Cytochrome P450 Family 2, Subfamily f (*Cyp2f2*). This evidential immaturity in the somatotropes suggested that the early ablation of LEPR resulted in the loss of a crucial signal for the maturation of somatotrope progenitor cells (13).

Our findings and hypothesis for the mutant female somatotrope population are informed by the pioneering studies by Rizzoti et al. (7, 8), who demonstrated the transition from *Sox2*+ to hormone-bearing *Sox9*+ progenitor cells using genetic lineage tracing. They determined that these progenitor cells persist in the adult and demonstrated their regenerative potential through their proliferative response to estradiol administration (in males). These cells could differentiate into new somatotropes, self-renew, and give rise to all pituitary hormone-bearing cell types *in vivo*. They also discovered they could activate them by classic endocrine physiological approaches, such as removing a target organ (8). Activation by adrenalectomy or gonadectomy (8) resulted in the presence of all pituitary hormone-bearing cell types in the Sox9+ progenitor cell population. However, lactotropes and gonadotropes outnumbered corticotropes after adrenalectomy, and somatotropes outnumbered gonadotropes after gonadectomy. The pituitary then adapted so that, eventually, only the required cell type was retained (corticotropes in the adrenalectomy model and gonadotropes in the gonadectomy model). This illustrated an essential adaptive response by the pituitary to maintain cell types based on physiological needs.

The presence of multihormonal expression in the mutant somatotropes suggests the potential for increased plasticity in the mutant somatotrope population. Our hypothesis postulates a role for leptin in promoting differentiation and maturation of cells in the progenitor cell population. It may play a role in the pituitary's adaptive responses. One mechanism may be through the leptin stimulation of common transcription factors. For example, we know that leptin stimulates Pou1f1 proteins, but not mRNA in female pituitaries (32). This transcription factor would promote the production of *Tshb, Prl, Gh* and *Ghrhr* (32).

The present study expands the transcript profile of other cell types in the somatotrope LEPR-null mutant pituitaries. Here we report that multihormonal expression in the deletion mutants on a control diet is also seen in lactotropes, thyrotropes, and corticotropes. These findings imply that the initial ablation of LEPR in early progenitor cells expressing the GH promoter may have impacted a Sox9+ progenitor cell with a broader fate and functions. Their presence may reflect the highly proliferative nature of Sox9+ cells, which migrate in the developing pituitary and develop further under the influence of specific releasing hormones in the vascular system (7, 8, 87, 88). Their developmental pattern would lead to gene expression that would promote their clustering with their cell type in the UMAP plot. Fate-mapping studies of somatotrope LEPR-null mutants may help verify this hypothesis.

Finally, scRNA-seq studies by Ho et al. (53) demonstrated distinct multihormonal populations in normal mouse populations, one of which contained *Gh, Prl, Pomc, Lhb*, and *Tshb*. It is unclear if this population is equivalent to the Sox9+ progenitor cell population descrobed by Rizzoti et al. (7, 8) Ho et al. were able to show a shift out of the multihormonal cluster as they stimulated the pituitary to support lactation. This points to the potential importance of these multihormonal cells in providing support for physiological functions. Future studies of FACS-purified LEPR-null mutant somatotropes may provide insight into the upstream regulators required to promote the maturation of these cells.

### 4.5 The impact of HFD on pituitary plasticity

An important novel finding in this study is that, in the mutant, the HFD reverses the upregulation of multihormonal expression in somatotropes, corticotropes, lactotropes, and thyrotropes. This reversal leads to reductions in expression of *Pomc, Prl, Lhb, Tshb*, and *Cga* in mutant somatotropes; *Lhb, Tshb, Cga*, and *Pomc* in lactotropes; *Prl* in corticotropes; and *Lhb* and *Prl* in thyrotropes. Additionally, the expression of progenitor cell markers in some of these cells is also reduced, especially in the somatotrope population. There are several possible explanations for these findings. Mechanisms underlying the HFD-induced reversal in hormone transcript expression may involve elevated serum levels of two cytokines (leptin and IL-6), which are reported to be trophic for pituitary cells (69, 75, 89). This would assume that the reduced multihormonal transcripts are the result of adaptive differentiation driven by IL-6 (34, 35, 58) or leptin (16), perhaps driving the cells to fully differentiate. An argument against this, however, is the lack of upregulated DEGs targeted by the JAK/STAT3 pathway and leptin in the HFD-exposed mutants [**Supplementary Table S19** (57)].

Alternatively, the loss of multihormonal expression may be the result of damage from the inflammatory and oxidative stress associated with the HFD and obesity (10). Aging alone promotes the inflammatory/immune responses of pituitary stem cells and renders them non-responsive to activation and regeneration (10). These multihormonal progenitor cells may have been deactivated by the HFD which causes oxidative stress. This would be expected to compromise pituitary plasticity. Future studies examining the impact of HFD and oxidative stress on pituitary stem cells in both young and aging females are necessary to test this hypothesis.

### 4.6 Summary

Our findings indicate an age-related vulnerability of the pituitary in 10-month-old female mice to HFD, including a greater number of DEGs in multiple pituitary cell populations compared to younger females on the HFD. While these older control females gained weight similarly to their younger counterparts on the HFD, older somatotrope LEPR-null mutant females displayed partial resistance to weight gain following the HFD and increased glucose tolerance. This response may be due to their behavior (lower food consumption and higher activity). The HFD reduced serum levels of growth hormone (GH) and prolactin (PRL) in both controls and somatotrope LEPR-null mutants. However, transcriptional changes were different when controls and mutants were compared. Translational regulatory pathways were upregulated in somatotropes and lactotropes of control females on the HFD, whereas these pathways were downregulated in mutant female somatotropes and lactotropes on the HFD. Lactotropes exhibited the highest number of DEGs in mutant females on a HFD, representing multiple signaling pathways and upstream regulators, indicating that these cells are significantly affected by the oxidative stress of obesity. Finally, our findings for multihormonal expression and immaturity in somatotropes in previous studies (13) have been expanded by new data showing that mutants on a control diet also have multihormonal expression and some progenitor cell genes in lactotropes, thyrotropes, and corticotropes. We also show that the HFD reduced this multihormonal expression and the expression of progenitor cell genes in all of these clusters. These findings suggest that the diet and/or obese state may have compromised progenitor cell functions, thus potentially reducing pituitary plasticity.

## Data Availability

The datasets presented in this study can be found in online repositories. The names of the repository/repositories and accession number(s) can be found in the article/**Supplementary Material**.
