# Supplementary material for: High fat diet-induced loss of pituitary plasticity in aging female mice with ablated leptin signaling in somatotropes
Source: Front Endocrinol (Lausanne). 2025 Jul 16;16:1617109. doi: 10.3389/fendo.2025.1617109 (PMC12307167; doi:10.3389/fendo.2025.1617109)
Supplement: Supplementary file 2 [file Presentation1.pptx]

## Slide 1
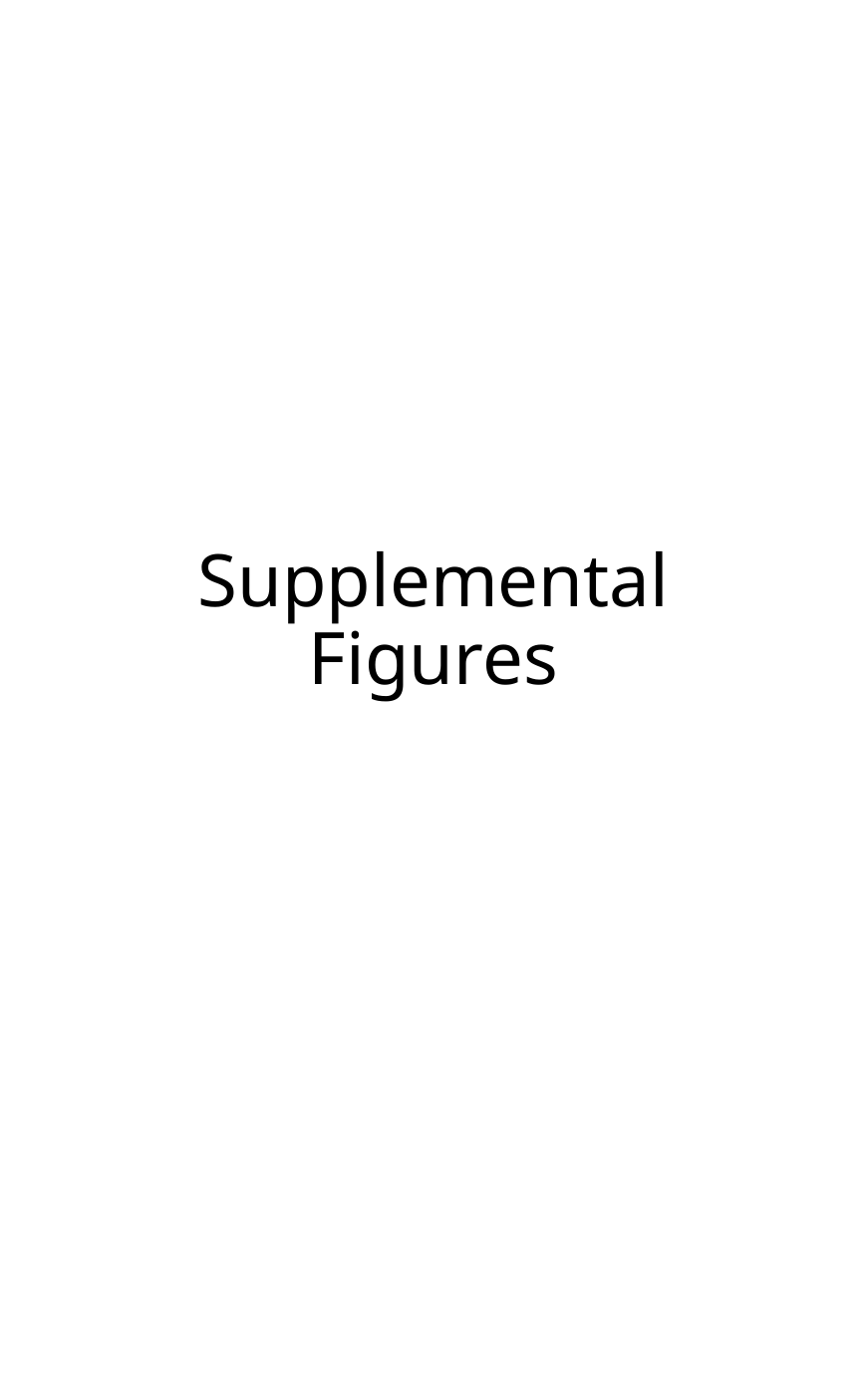

# Supplemental Figures

## Slide 2
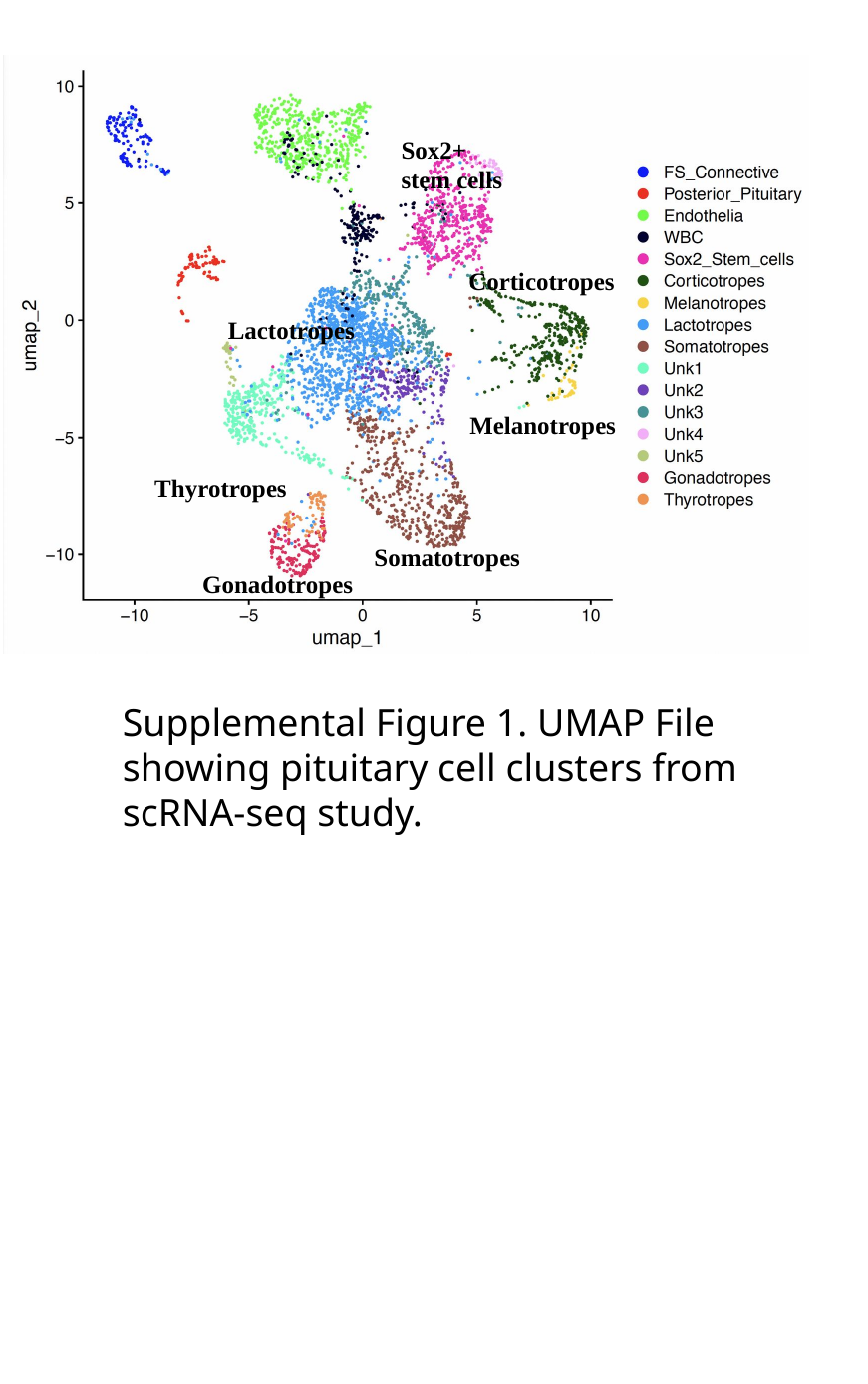

Sox2+
stem cells
Corticotropes
Lactotropes
Melanotropes
Thyrotropes
Somatotropes
Gonadotropes
Supplemental Figure 1. UMAP File showing pituitary cell clusters from scRNA-seq study.
