# Supplementary material for: High fat diet-induced loss of pituitary plasticity in aging female mice with ablated leptin signaling in somatotropes
Source: Front Endocrinol (Lausanne). 2025 Jul 16;16:1617109. doi: 10.3389/fendo.2025.1617109 (PMC12307167; doi:10.3389/fendo.2025.1617109)
Supplement: Supplementary file 3 [file SupplementaryFile1.pdf]

Table S2. Somatotrope Cluster: HFD

Table S2A. Female Somatotropes Controls HFD vs CD Avg_log2FC ≤0.58; adj p<0.055		
gene	avg_log2FC	p_val_adj
AY036118	1.155662465	2.36E-13
Lars2	0.791342604	0.006155505
Rps27	0.78782812	9.32E-09
Rpl38	0.783617311	2.46E-10
Rps21	0.749736177	2.28E-19
Rpl41	0.740407289	2.24E-11
Rps29	0.735017484	1.88E-09
Gm42418	0.710309294	1.44E-12
Rpl37a	0.705390072	1.00E-09
Rps28	0.638720461	0.000305138
Rpl37	0.624025532	5.26E-08
Rpl35a	0.587231225	0.000118526
Rplp2	0.548908668	0.006721495
**Table S2B Female Mutants HFD vs CD Avg_log2FC ≤0.58; adj p<0.055**		
Hspa1a	1.726227052	0.002349738
Hspa1b	1.468154155	3.27E-07
Dnaja1	1.137234882	1.40E-05
Mt2	1.056448353	4.50E-05
Gh	0.619860562	3.42E-06
Rpl32	-0.553203912	0.004081137
Rpl23	-0.554242717	2.00E-05
Dusp1	-0.577926341	0.020128799
Rpl36a	-0.585194342	0.042325558
Rpl24	-0.623394889	1.55E-05
Fos	-0.636389953	4.53E-06
Igfbp5	-0.715602653	0.014268915
Rpl7a	-0.782039248	0.012136338
Cox8a	-0.787886005	0.010261472
Atp5l	-0.802992897	0.015379338
Cst3	-0.83274024	2.18E-08
Cga	-0.843487675	1.32E-06
Rpl36	-0.871314268	1.72E-05
Pomc	-0.88829364	3.64E-08
Rps18	-0.915149756	1.22E-05
Klf4	-0.933462829	1.67E-07
Atp5h	-1.088579145	0.00331362
Actb	-1.223239063	2.52E-11
Uqcr11	-1.275611948	0.00655533
Syt4	-1.450137973	0.002713605
Prl	-1.522226727	2.56E-35
Mia	-2.537284357	0.000420759
Cd74	-2.544759478	1.83E-08
H2-Aa	-2.809656627	0.000691347
Phpt1	-3.484816937	0.027636416

Supplemental Table S2C Gene Ontology Analysis of 45 down-regulated genes in Mutant Female Somatotrope Cluster: Comparing HFD vs CD, adjusted p<0.055		
GO Term (GO ID)	Genes annotated to the GO Term	Go Term Usage in Gene List
Cytoplasmic translation (GO:002181)	*Rpl17, Rpl21, Rpl23, Rpl24, Rpl26, Rpl27a, Rpl32, Rpl36, Rpl36a, Rpl41, Rpl9, Rps10, Rps12, Rps14, Rps18, Rps20, Rps23, Rps29, Rps3a1, Rps8*	20/45 genes
Signaling (GO:0023052)	*Cd74, Cga, Dusp1, Egr1, Fos, Igfbp5, Klf4, Phpt1, Pomc, Prl, Rpl23, Rpl24, Rpl26, Rps12, Rps20, Syt4, Tpt1*	17/45 genes
Cell differentiation (GO:0030154)	*Actb, Cd74, Egr1, Fos, H2-Aa, H3f3b, Ier2, Igfbp5, Klf4, Rpl24, Rps14, Rps3a1, Syt4*	13/45 genes
Regulation of DNA-templated transcription (GO:0006355)	*Actb, Cd74, Egr1, Fos, Fosb, Ier2, Klf4, Pomc, Prl, Rpl10, Rpl23, Rps14*	12/45 genes
Programmed cell death (GO:0012501)	*Actb, Cd74, Dusp1, Egr1, Klf4, Pomc, Rpl10, Rpl26, Rps3a1, Tpt1*	10/45 genes
ribosome biogenesis (GO:0042254)	*Rpl10, Rpl24, Rpl26, Rps12, Rps14, Rps23, Rps3a1, Rps8*	8/45 genes
Cell Motility (GO:0048870)	*Actb, Cd74, Dusp1, Egr1, Ier2, Igfbp5, Klf4, Phpt1*	8/45 genes
Protein containing complex assembly	*Cd74, H2-Aa, H3f3b, Rpl10, Rpl24, Rps14*	6/45 genes
Cell adhesion (GO:0007155)	*Actb, Cd74, Dusp1, H2-Aa, Klf4, Mia*	6/25 genes

Supplemental Table S2: Differentially expressed genes in somatotrope cluster. Table S2A from control female on a HFD. Table S2B from somatotrope LEPR-null mutant female on a HFD. Red=upregulated genes and blue=down-regulated genes. Yellow highlight indicates genes important for hormone production in pituitary. Table S2C Gene Ontology Analysis of Down-regulated DEGs from somatotrope LEPR-null mutant female on a HFD. The yellow highlight designates genes important to pituitary cell hormone production.

Supplemental Table S3A. DEGs in Female Lactotrope cluster Control HFD vs CD; Avg_log2FC ≤0.58; adj p<0.055		
gene	avg_log2FC	p_val_adj
Snhg18	2.071769034	8.67E-06
Zfos1	0.930431952	0.000223896
Rps29	0.915561337	3.63E-26
Rps21	0.868378706	1.05E-36
Rps27	0.822912049	9.75E-08
Gm10076	0.793283032	0.050462262
Gm42418	0.792474914	1.19E-25
mt-Nd3	0.774451825	2.54E-05
Rpl38	0.741522501	9.11E-20
Rpl37	0.719285971	1.95E-17
Rps28	0.689997082	1.93E-09
Rpl37a	0.673291758	1.55E-14
Rpl39	0.630106279	7.50E-07
Rpl36	0.599965491	1.43E-07
Rpl35a	0.584727912	4.07E-06
Hspa5	-0.500642366	0.032211757
Pou1f1	-0.751868667	1.55E-06
Spcs2	-0.771768224	0.000112984
Cga	-1.121477042	0.013496701

Supplemental Table S3B Gene Ontology Analysis of 22 up-regulated genes in Control Female Lactotrope Cluster: HFD vs CD; adjusted p<0.055		
GO Term (GO ID)	Genes annotated to the GO Term	Go Term Usage in Gene List
cytoplasmic translation (GO:0002181)	*Rpl30, Rpl34, Rpl35a, Rpl36, Rpl37a, Rpl38, Rpl39, Rpl41, Rplp2, Rps21, Rps23, Rps28, Rps29*	13/22 genes
ribosome biogenesis (GO:0042254)	*Rpl35a, Rpl38, Rps21, Rps23, Rps27, Rps28*	6/22 genes
protein-containing complex assembly (GO:0065003)	*Rpl38, Rps27, Rps28, mt-Nd4*	4/22 genes
transmembrane transport (GO:0055085)	*Sec61g, Zfos1, mt-Nd4*	3/22 genes

Table S3C. Upregulated DEGs in Female Lactotrope cluster Mutant (LEPR-null Somatotrope). HFD vs CD
Avg_log2FC ≤0.58; adj p<0.055

gene	avg_log2FC	p_val_adj
Dnaja4	3.690586404	6.65E-18
Hspa1b	3.164517592	3.93E-55
Hspa1a	2.805155464	1.06E-64
Phlda1	2.750553926	1.41E-11
Dnajb1	2.436391295	8.78E-45
Hspb1	2.406888044	3.42E-19
Bag3	2.246979753	3.27E-09
Serpinh1	2.093733492	2.64E-13
Serpine1	1.945810218	0.028679889
Dnajb4	1.800372174	1.84E-07
Crhbp	1.631025437	5.58E-32
Arc	1.5249645	3.54E-14
Creb3l1	1.489836276	0.000196975
Gal	1.432169026	5.09E-30
Hsph1	1.410478485	1.69E-05
Srxn1	1.35900637	0.003386696
Npas4	1.324020788	1.80E-10
Rgs2	1.227614428	5.96E-16
Dnaja1	1.182629341	2.12E-22
Gm10076	1.166207129	1.45E-32
Gm10260	1.127832487	8.13E-24
Rnd3	1.104960748	0.021337868
Rhob	1.087781158	0.035901175
Pde10a	1.080128895	5.63E-10
Nr4a2	1.043696154	3.82E-09
Pak3	0.983945347	0.000470903
Aebp1	0.945645592	2.79E-16
Peg3	0.933088977	4.01E-08
Hsp90aa1	0.917232153	8.35E-18
Jun	0.855591004	1.08E-38
Ascl1	0.834040719	0.004962601
Rtn1	0.826487823	0.02461833
Gpx3	0.797672351	7.38E-06
Zfp36l2	0.751022258	2.60E-10
Cited2	0.747334821	6.29E-06
Hsp90ab1	0.723253171	1.58E-22
Atf3	0.716209352	3.95E-17
Chgb	0.709970946	1.18E-18
Hspa5	0.704754235	1.28E-10
Pdia3	0.674430035	5.07E-12
Fam46a	0.664454237	1.35E-06
Calb1	0.656403645	0.001880281
Txnip	0.647803393	0.000100178
Meg3	0.647014107	1.34E-39
Sox4	0.646075708	0.000109042
Cyr61	0.631515163	0.000289107
Stmn1	0.620676456	0.001114674
Camk2n1	0.619581986	0.000814619
Six6	0.60374421	0.000104675
Nap1l5	0.597815556	1.99E-10
Gadd45g	0.592754589	0.000177709
Hspa8	0.590325265	4.35E-11
Itm2b	0.577196638	3.98E-31

Supplemental Table S3D. Downregulated DEGs in Female Lactotrope cluster Mutant (LEPR-null Somatotrope). HFD vs CD;
Avg_log2FC ≤0.58; adj p<0.055

gene	avg_log2FC	p_val_adj
Zfp36	-0.577273694	7.83E-11
Cox6c	-0.597849875	0.000120697
Atp5g2	-0.653484574	0.001652651
Rpl23a	-0.670717598	0.004077807
Cox4i1	-0.673833219	7.42E-07
Shfm1	-0.687391579	0.043117513
Socs3	-0.722067319	4.09E-05
mt-Nd3	-0.730300171	1.34E-06
Mcee	-0.731215877	2.20E-05
2410015M20Rik	-0.738121371	0.006865871
Psmb3	-0.786187111	0.004150249
Tceb2	-0.786572807	0.014108616
Ndufb3	-0.798108137	0.008274025
Atp5f1	-0.79920988	0.038952324
Cd81	-0.960034568	0.001219667
Igfbp5	-0.997081671	1.45E-05
Suclg1	-1.000947761	0.001397611
Coa3	-1.081207333	0.037275676
Cox5a	-1.11342249	0.001897399
Kdelr2	-1.114017907	0.000145472
Rhoa	-1.130801577	0.005449418
Cbx3	-1.174484016	0.007326683
Pgls	-1.176231678	0.019740477
Lamtor4	-1.208073426	0.001677007
Cga	-1.269964325	1.43E-64
Mrpl30	-1.306471775	0.018396068
Pomc	-1.320760378	3.65E-71
Ndufs3	-1.3842764	0.004901759
Actb	-1.401103629	8.23E-48
Rps18-ps3	-1.403274208	0.001194581
0610012G03Rik	-1.426792015	0.017164982
Chchd10	-1.440080936	1.35E-05
H2-D1	-1.449875628	1.07E-18
Bri3	-1.489985842	0.000205803
Erdr1	-1.510717283	0.015246092
Sarnp	-1.523094153	0.001029196
Atox1	-1.571181924	0.002564975
Phpt1	-1.667903634	0.003416417
Esd	-1.697878693	0.00470606
Capza2	-1.770942155	0.000812919
Bcl7c	-1.868497465	0.000156811
Ifitm3	-1.888797026	1.98E-07
H2-K1	-1.919456263	4.08E-10
Apoe	-1.926881663	5.74E-26
B2m	-2.028524005	4.90E-15
C1qa	-2.050700796	0.008646707
Lhb	-2.080785282	4.47E-20
Apod	-2.12457911	0.000170583
Gstm1	-2.170105575	9.58E-06
Klf2	-2.396876437	2.22E-05
Nfia	-2.404071939	3.11E-05
Rbpms	-2.425445589	0.005113615
Tmsb4x	-2.431257894	3.81E-52
Mia	-2.758644797	1.01E-08
Tshb	-3.223340182	1.43E-17
Cd74	-4.957591392	3.06E-40

Supplemental Table S3E Gene Ontology Analysis of 77 up-regulated genes in Mutant Female Lactotrope Cluster: HFD vs CD; adjusted p<0.055		
GO Term (GO ID)	Genes annotated to the GO Term	Go Term Usage in Gene List
Signaling (GO:0023052)	*Arc, Ascl1, Atf3, Bag3, Calb1, Camk2n1, Cited2, Creb3l1, Crhbp, Cyr61, Dbi, Dlk1, Dnaja1, Dusp1, Gadd45g, Gh, Hspa5, Hspa8, Hspb1, Jun, Klf4, Nnat, Npas4, Nr4a1, Nr4a2, Pak3, Pcsk1n, Pde10a, Pdia3, Rgs2, Rhob, Rnd3, Serpine1, Sox4, Stmn1, Txnip, Ubb, Zfp36l2*	38/77 genes
cell differentiation (GO:0030154)	*AY036118, Arc, Ascl1, Atf3, Btg2, Cited2, Creb3l1, Cyr61, Dbi, Dlk1, Fam46a, Gadd45g, Gh, Hsp90aa1, Hspa5, Hspb1, Jun, Klf4, Manf, Meg3, Npas4, Nr4a1, Nr4a2, Pak3, Rgs2, Rhob, Rtn1, Serpine1, Serpinh1, Sox4, Stmn1, Txnip, Ubb, Zfp36l2*	34/77 genes
programmed cell death (GO:0012501)	*Ascl1, Atf3, Bag3, Btg2, Cited2, Creb3l1, Cyr61, Dnaja1, Dusp1, Gadd45g, Hsp90aa1, Hsp90ab1, Hspa1b, Hspa5, Hspb1, Hsph1, Jun, Klf4, Nr4a1, Nr4a2, Pak3, Pdia3, Peg3, Phlda1, Rhob, Serpine1, Sox4, Txnip, Ubb*	29/77 genes
protein maturation (GO:0051604)	*Aebp1, Dnaja1, Dnaja4, Dnajb1, Dnajb4, Hsp90aa1, Hsp90ab1, Hsp90b1, Hspa1a, Hspa1b, Hspa5, Hspa8, Hspb1, Hspe1, Hsph1, Pcsk1n, Pdia3, Serpine1, Serpinh1, Sox4*	20/77 genes
regulation of DNA-templated transcription (GO:0006355)	*Aebp1, Ascl1, Atf3, Btg2, Cited2, Creb3l1, Cyr61, Dnajb1, Dnajb4, Hspa8, Jun, Klf4, Npas4, Nr4a1, Nr4a2, Peg3, Six6, Sox4, Txnip*	17/77 genes
cell motility (GO:0048870)	*Arc, Ascl1, Cited2, Cyr61, Dnaja1, Dnaja4, Dusp1, Hsp90aa1, Hspa5, Hspb1, Jun, Klf4, Nr4a1, Nr4a2, Pak3, Rhob, Rnd3, Serpine1, Stmn1*	19/77 genes
protein folding (GO:0006457)	*Dnaja1, Dnaja4, Dnajb1, Dnajb4, Hsp90aa1, Hsp90ab1, Hsp90b1, Hspa1a, Hspa1b, Hspa5, Hspa8, Hspb1, Hspe1, Hsph1, Pdia3*	15/77 genes

Supplemental Table S3F Gene Ontology Analysis of 77 downregulated genes in Mutant Female Lactotrope Cluster: HFD vs CD; adjusted p<0.055		
GO Term (GO ID)	Genes annotated to the GO Term	Go Term Usage in Gene List
Signaling (GO:0023052)	*Apod, Apoe, Cd74, Cd81, Cga, Chchd10, Fos, Ifitm3, Igfbp5, Jund, Klf2, Lamtor4, Lhb, Mt1, Ndufs3, Ndufs6, Nfia, Pfdn5, Phpt1, Pomc, Ppia, Prl, Rbpms, Rhoa, Rpl24, Rps19, Rps20, Socs3, Tpt1, Tshb, Zfp36*	31/77 genes
cell differentiation (GO:0030154)	*Actb, Apoe, B2m, Bcl7c, C1qa, Cd74, Cd81, Fos, H2-D1, H2-K1, H3f3b, Ier2, Igfbp5, Junb, Jund, Klf2, Ndufs6, Nfia, Ppia, Rhoa, Rpl24, Rps19, Socs3, Tmsb4x, Zfp36*	25/77 genes
regulation of DNA-templated transcription (GO:0006355)	*Actb, Apoe, Bcl7c, Cbx3, Cd74, Cd81, Chchd10, Chchd2, Fos, Fosb, Ier2, Junb, Jund, Klf2, Nfia, Pfdn5, Pomc, Ppia, Prl, Rhoa, Sarnp, Tmsb4x, Zfp36*	23/77 genes
protein-containing complex assembly (GO:0065003)	*Apoe, Atp5g2, B2m, Capza2, Cd74, Chchd10, Coa3, Fau, H3f3b, Ndufb3, Ndufs3, Ndufs6, Pfdn5, Rbpms, Rhoa, Rpl23a, Rpl24, Rps19, Shfm1, Tmsb4x, mt-Nd5*	21/77 genes
generation of precursor metabolites and energy (GO:0006091)	*Atp5f1, Chchd10, Chchd2, Cox4i1, Cox5a, Ndufb3, Ndufs3, Ndufs6, Pgls, Pomc, Rhoa, Suclg1, mt-Co1, mt-Nd3, mt-Nd5*	15/77 genes
cell motility (GO:0048870)	*Actb, Apod, Apoe, Cd74, Cd81, Erdr1, Ier2, Igfbp5, Phpt1, Ppia, Rhoa, Rps19, Tmsb4x*	13/77 genes
programmed cell death (GO:0012501)	*Actb, Apoe, Atox1, Bcl7c, Cd74, Chchd10, Mt1, Ndufs3, Pomc, Ppia, Rhoa, Tpt1, Zfp36*	13/77 genes

Supplemental Table S3: Differentially expressed genes in Lactotrope cluster from control female on a HFD. Red=upregulated genes and Blue=downregulated genes. Yellow highlight indicates genes important for hormone production in pituitary. Table S3B Gene Ontology Analysis of Up-regulated DEGs from lactotrope cluster from control female on a HFD. Table S3C Upregulated DEGs in lactotrope cluster from somatotrope LEPR-null mutants on a HFD. Table S3D Down regulated DEGs in lactotrope cluster from somatotrope LEPR-null mutant on a HFD. Table S3E and S3F. Gene ontology analysis of up- and down- regulated genes from lactotrope cluster in somatotrope LEPR-null mutant on a HFD. Highlighted genes are those most important to pituitary cell hormone production.

Supplemental Table S4A. DEGs in Female Corticotrope cluster, Mutant (Som LEPR-null); HFD vs CD Avg_log2FC ≤0.58; adj p<0.055		
gene	avg_log2FC	p_val_adj
Hspa1b	2.528194943	0.027360298
Hspa1a	2.151819064	0.00349672
Srxn1	1.755160095	0.025922856
Gm10076	1.279975416	7.80E-07
Mt1	1.141934476	4.32E-15
Gm10260	1.081429737	0.000502858
Nnat	0.993895498	1.02E-10
Fth1	0.93041938	2.26E-20
Calm1	0.786982378	0.000132413
Rps28	0.752473938	8.68E-05
AY036118	0.729525749	3.62E-09
Rps29	0.664825396	2.13E-07
Rpl37	0.633259481	1.89E-06
Rpl38	0.620785486	0.002035177
Rpl7	0.575000638	0.01370074
Naca	0.55871339	0.023094244
Kcnq1ot1	-0.641730058	2.16E-05
Btg2	-0.642726051	8.99E-09
Chgb	-0.80658407	2.43E-05
G0s2	-0.811655059	0.042488508
Ier2	-0.897230791	6.01E-05
Junb	-1.010222618	1.51E-14
Egr1	-1.021425396	1.61E-06
Sat1	-1.052663159	0.009688887
Dlk1	-1.079466606	9.85E-08
Actb	-1.177354032	3.88E-13
Nr4a1	-1.2386652	9.79E-06
Luc7l2	-1.2416429	0.007152161
Pde10a	-1.244614467	0.038592626
Malat1	-1.288229057	5.90E-16
Prl	-1.387380013	3.92E-51
Syt4	-1.418560113	0.000181828
Meg3	-1.50197056	2.54E-16
C2cd4b	-1.539575514	0.007849467
Fam46a	-1.768065791	8.98E-05
Ddx21	-1.811655059	8.33E-17
Fos	-1.832542426	1.71E-31
Kdelr2	-2.002796547	0.005236392
Pam	-2.005134789	8.72E-08
Dusp1	-2.090878703	1.03E-07
Fosb	-2.107704523	3.78E-17
Cd74	-2.692073444	3.92E-10
Xist	-2.847278969	9.21E-15
B2m	-2.864122479	3.57E-11
Klf4	-3.046871521	5.96E-05
Klf2	-3.355975576	0.00010857
Tsix	-3.39661756	0.009151725
Slc24a5	-3.927132277	0.005649069

Supplemental Table S4B. DEGs in Female Thyrotrope cluster, Mutant Som LEPR null) HFD vs CD Avg_log2FC ≤0.58; adj p<0.055.		
gene	avg_log2FC	p_val_adj
Chgb	1.482239556	0.005077134
Hsp90aa1	1.42675092	0.01810036
AY036118	1.004127886	0.021045477
Rps29	0.921311111	0.00119553
Rps17	0.918058386	0.006717152
Eef1a1	0.776981273	0.000134942
Deb1	-1.067114196	1.68E-05
Btg2	-1.312939312	0.00502415
Fos	-1.418995513	0.004546061
Junb	-1.472752997	0.000461793
Prl	-1.747291519	1.31E-15
Actb	-1.777607579	0.005218088
Fosb	-1.989658056	0.014748834
Lhb	-3.780337371	2.13E-10
Spp1	-3.94753258	0.006599397
Klf4	-4.050626073	0.004721083

Supplemental Table S4C Gene Ontology Analysis of 26 up-regulated genes in Mutant Female Corticotrope Cluster: HFD vs CD; adjusted p<0.055		
GO Term (GO ID)	Genes annotated to the GO Term	Go Term Usage in Gene List
cytoplasmic translation (GO:0002181)	*Rpl13, Rpl13a, Rpl19, Rpl22l1, Rpl27a, Rpl37a, Rpl38, Rpl7, Rps19, Rps28, Rps29*	11/26 genes
signaling (GO:0023052)	*Calm1, Gnas, Mt1, Nnat, Rpl37, Rps19*	6/26 genes
protein-containing complex assembly (GO:0065003)	*Rpl13a, Rpl38, Rps19, Rps28, mt-Nd4*	5/26 genes
cell differentiation (GO:0030154)	*AY036118, Gnas, Naca, Rps19*	4/26 genes
ribosome biogenesis (GO:0042254)	*Rpl38, Rpl7, Rps19, Rps28*	4/26 genes
programmed cell death (GO:0012501)	*Hsp90ab1, Hspa1b, Mt1, Naca*	4/26 genes
protein folding (GO:0006457)	*Hsp90ab1, Hspa1a, Hspa1b*	3/26 genes

Supplemental Table S4D Gene Ontology Analysis of 36 Downregulated genes in Mutant Female Corticotrope Cluster: HFD vs CD; adjusted p<0.055		
GO Term (GO ID)	Genes annotated to the GO Term	Go Term Usage in Gene List
cell differentiation (GO:0030154)	*Actb, B2m, Btg2, Cd74, Dlk1, Egr1, Fam46a, Fos, H2-Aa, Ier2, Junb, Kcnq1ot1, Klf2, Klf4, Meg3, Nr4a1, Slc24a5, Syt4, Tmsb4x, Tsix, Tyrobp, Xist*	22/36 genes
regulation of DNA-templated transcription (GO :0006355)	*Actb, Btg2, Cd74, Ddx21, Egr1, Fos, Fosb, Ier2, Junb, Klf2, Klf4, Nr4a1, Prl, Tmsb4x, Tsix*	15/36 genes
signaling (GO:0023052)	*Cd74, Ddx21, Dlk1, Dusp1, Egr1, Fos, G0s2, Klf2, Klf4, Nr4a1, Pde10a, Prl, Scg5, Syt4, Tyrobp*	15/36 genes
cell motility (GO:0048870)	*Actb, Cd74, Dusp1, Egr1, Ier2, Kcnq1ot1, Klf4, Nr4a1, Tmsb4x*	9/36 genes
programmed cell death (GO:0012501)	*Actb, Btg2, Cd74, Dusp1, Egr1, G0s2, Kcnq1ot1, Klf4, Nr4a1*	9/36 genes
DNA-templated transcription (GO:0006351)	*Ddx21, Egr1, Fos, Fosb, Junb, Klf4, Nr4a1, Tsix*	8/36 genes
reproductive process (GO:0022414)	*Egr1, Junb, Kcnq1ot1, Meg3, Prl, Tsix, Xist*	7/36 genes
cell adhesion (GO:0007155)	*Actb, B2m, C2cd4b, Cd74, Dusp1, H2-Aa, Klf4*	7/36 genes

Supplemental Table S4A. DEGs in corticotrope cluster from somatotrope LEPR-null females after a HFD.
Supplemental Table S4B. DEGs in thyrotrope cluster from somatotrope LEPR-null females after a HFD. Red=upregulated genes and blue=down-regulated genes. Yellow highlight indicates genes important for hormone production in pituitary.
Supplemental Table S4C. Gene Ontology analysis of upregulated DEGs in somatotrope LEPR-null mutant female corticotrope cluster. S4D Gene ontology analysis of downregulated genes in somatotrope LEPR-null mutant female corticotrope cluster. The yellow highlight designates important genes involved in pituitary hormone production.

Supplemental Table S5. DEGs in Control Female Gonadotrope cluster, HFD vs CD Avg_log2FC ≤0.58; adj p<0.055		
gene	avg_log2FC	p_val_adj
AY036118	1.10115567	6.27E-09
Gm42418	0.89987703	4.37E-07
Lhb	0.85120154	0.00010345
Rpl37	0.70309788	0.00988804
Prl	-0.633188	0.00108763
Nnat	-1.3027009	1.54E-08
Fshb	-1.7404897	6.21E-21
Rbp4	-2.0199706	0.00159381

Supplemental Table S5
DEGs in Control female gonadotrope cluster on a HFD (vs CD).
Red=upregulated genes and blue=downregulated genes. Yellow highlight designates those genes most important to pituitary cell function.

Supplemental Table S6A. DEGs in Female Sox2 Stem cell cluster Control-HFD vs CD
Avg_log2FC ≤0.058; adj p<0.055

gene	avg_log2FC	p_val_adj
AY036118	1.16908686	7.75E-11
Lars2	1.01825242	0.00543564
Gm42418	0.96053555	1.17E-19
Rpl37	0.73047539	1.45E-08
Rpl35a	0.57371092	0.0204152
Rps29	0.56642998	4.73E-05
Rpl38	0.55799188	0.04555196
Jun	-0.7783425	0.01686307
Plat	-1.020739	0.02827896
B2m	-1.3734969	9.96E-13
H2-D1	-1.6473595	1.07E-23
H2-K1	-1.7601898	1.52E-20
H2-Ab1	-2.2222956	1.42E-09
H2-Aa	-2.3174073	6.82E-17
Cd74	-2.6819593	6.27E-32
Iigp1	-2.997229	0.03741726

Supplemental Table S6B. DEGs in Female Sox2 Stem cell cluster Mutant, HFD vs CD
Avg_log2FC ≤0.058; adj p<0.055

gene	avg_log2FC	p_val_adj
Lcn2	1.95980336	0.00278604
Mt2	0.7864403	3.02E-06
Igfbp5	0.76880423	9.64E-14
AY036118	0.76867121	1.25E-17
Prdx1	0.75275334	1.37E-08
Hspb1	0.74339453	0.00014481
Hspa1a	0.72866229	0.03700322
Apoe	0.66688865	0.0310045
Mt1	0.66083136	4.88E-08
App	0.64694939	0.00167211
Gstm1	0.63898982	0.00030271
Hsp90ab1	0.59855722	1.16E-11
Gh	0.58575729	1.70E-06
Rpl34	-0.6067093	6.90E-09
Fosb	-0.6579905	4.89E-08
Dusp1	-0.7364159	2.53E-06
Rps18	-0.7472575	4.20E-07
Eif3k	-0.8258979	0.03473722
Bex1	-0.9374896	0.00535669
Chgb	-1.0678043	2.30E-13
Klf4	-1.1399825	3.28E-06
Scg5	-1.1878709	1.26E-13
Meg3	-1.1927	7.02E-09
H2-D1	-1.2072781	2.29E-15
Crhbp	-1.228269	0.04744176
Nfkbiz	-1.2419439	0.00513986
B2m	-1.2723915	1.38E-11
Pcsk1n	-1.3446482	8.78E-13
Nr4a2	-1.3595135	0.00020065
Prl	-1.384506	2.15E-33
Scg2	-1.4238198	1.98E-07
H2-K1	-1.4558305	1.64E-13
Nnat	-1.4601842	8.60E-19
Dlk1	-1.4856251	2.85E-13
Fam183b	-1.5237249	0.00172959
Calb1	-1.5547698	0.01437962
Psmb8	-1.5761923	0.00026038
Pam	-1.5839279	0.00046261
Gdpd3	-1.6433065	0.01628307
Nap1l5	-1.8803457	6.19E-05
Pou1f1	-1.8956936	0.00161717
H2-Aa	-1.927338	9.37E-14
Cd74	-1.9975706	4.05E-22
Plagl1	-2.058344	0.00217338
Kdelr2	-2.0939679	6.74E-05
1700086L19Rik	-2.0950025	7.33E-06
Resp18	-2.1236354	8.19E-28
H2-Ab1	-2.223452	3.06E-18
Fam46a	-2.2386856	2.97E-08
Irgm1	-2.357552	3.02E-06
H2-T23	-2.4506614	0.00036299
C2cd4b	-2.5308318	5.58E-05
Syt4	-2.563872	2.45E-10
5330416C01Rik	-2.6789304	0.00254736
Pde10a	-2.6964178	3.52E-06
Ascl1	-3.1668684	0.00030337
Iigp1	-3.4599993	8.93E-09
H2-Q7	-4.0356239	0.00014977

Supplemental Table S6C Gene Ontology Analysis of 77 downregulated genes in Mutant Female Sox2 Stem cell Cluster: HFD vs CD; adjusted p<0.055		
GO Term (GO ID)	Genes annotated to the GO Term	Go Term Usage in Gene List
anatomical structure development. (GO:0048856)	*Ascl1, B2m, Calb1, Cd74, Dlk1, Dusp1, Fam46a, H2-Aa, H2- Ab1, H2-D1, H2-K1, H2-Q7, H2-T23, Irgm1, Klf4, Meg3, Nfkbiz, Nnat, Nr4a2, Plagl1, Pou1f1, Prl, Resp18, Scg2, Syt4*	25/43 genes
Signaling GO:0023052)	*Ascl1, Calb1, Cd74, Crhbp, Dlk1, Dusp1, Iigp1, Irgm1, Klf4, Nfkbiz, Nnat, Nr4a2, Pcsk1n, Pde10a, Pou1f1, Prl, Scg2, Scg5, Syt4*	19/43 genes
cell differentiation (GO:0030154)	*Ascl1, B2m, Cd74, Dlk1, Fam46a, H2-Aa, H2-Ab1, H2-D1, H2-K1, Irgm1, Klf4, Meg3, Nfkbiz, Nr4a2, Plagl1, Pou1f1, Psmb8, Syt4*	18/43 genes
immune system process (GO:0002376)	*B2m, Cd74, Dlk1, Dusp1, H2-Aa, H2-Ab1, H2-D1, H2-K1, H2-Q7, H2-T23, Iigp1, Irgm1, Nfkbiz, Pou1f1, Psmb8, Scg2*	16/43 genes
defense response to other organism (GO:0098542)	*B2m, Cd74, H2-Aa, H2-Ab1, H2-K1, H2-Q7, Iigp1, Irgm1,Nfkbiz*	9/43 genes
cell adhesion (GO:0007155)	*B2m, C2cd4b, Cd74, Dusp1, H2-Aa, H2-Ab1, Irgm1, Klf4, Nfkbiz*	9/43 genes
regulation of DNA-templated transcription (GO:0006355)	*Ascl1, Cd74, Fosb, Klf4, Nfkbiz, Nr4a2, Plagl1, Pou1f1, Prl*	9/43 genes
protein-containing complex assembly (GO:0065003)	*B2m, Cd74, H2-Aa, H2-Ab1, Irgm1, Nap1l5, Nfkbiz*	7/43 genes

Supplemental Table S6A. DEGs in Sox2 Stem cell cluster from control females on a HFD.
Supplemental Table S6B. DEGs in Sox2 stem cell cluster from somatotrope LEPR null -mutant
Red=upregulated genes and blue=highlighted genes. Yellow highlight indicates genes important for hormone production in pituitary.
Supplemental Table S6C. Gene ontology analysis of down regulated genes in Sox2 stem cell cluster from somatotrope LEPR-null mutant. Yellow highlight indicates genes important for hormone production in pituitary.
