# Supplementary material for: High fat diet-induced loss of pituitary plasticity in aging female mice with ablated leptin signaling in somatotropes
Source: Front Endocrinol (Lausanne). 2025 Jul 16;16:1617109. doi: 10.3389/fendo.2025.1617109 (PMC12307167; doi:10.3389/fendo.2025.1617109)
Supplement: Supplementary file 4 [file SupplementaryFile2.pdf]

Direct Targets of Upstream Regulators.
Mutant Mice: HFD vs CD

Supplemental Table S7. Cyclic AMP Responsive Element Binding Protein1					
**Genes in the CREB1 network**	**Somatotropes**	**Lactotropes**	**Thyrotropes**	**Corticotropes**	**Sox2 Stem Cells**
SRXN1	1.858235	1.359006	N/A	1.75516	-0.49811
GAL	0.915721	1.432169	-0.61891	0.238718	N/A
BAG3	N/A	2.24698	N/A	N/A	N/A
LCN2	N/A	N/A	N/A	N/A	1.959803
NPAS4	0.521103	1.324021	-0.19265	-0.35222	N/A
HSPA5	0.299454	0.704754	0.074294	-0.08268	-0.34517
RGS2	0.349359	1.227614	N/A	N/A	-0.12407
TPT1	-0.34038	-0.05147	0.347031	0.179412	N/A
NNAT	0.160671	0.308295	0.186642	0.993895	-1.46018
CHGB	-0.34433	0.709971	1.48224	-0.80658	-1.0678
SAT1	1.103898	0.11545	-0.8365	-1.05266	0.082254
CGA	-0.84349	-1.26996	0.957144	N/A	N/A
JUN	0.107065	0.855591	-1.19265	-0.55421	-0.06548
ATF3	N/A	0.716209	-0.47275	-0.52487	N/A
COX8A	-0.78789	-0.44187	N/A	0.490103	-0.09311
CRHBP	-1.15314	1.631025	-0.29218	-1.10116	-1.22827
ARC	0.345452	1.524964	N/A	-1.48973	0.227707
SCG2	-0.51019	N/A	0.846349	-0.3295	-1.42382
CEBPB	0.098603	-0.14968	-1.51457	-0.28757	0.262057
CHGA	N/A	-0.24043	0.385528	0.057715	-1.20273
NAP1L5	-0.55842	0.597816	-1.16993	-0.36525	-1.88035
BTG2	0.192092	0.305083	-1.31294	-0.64273	N/A
ZFP36	N/A	-0.57727	-0.34792	-0.94596	0.124612
EGR1	-0.22039	N/A	-1.11464	-1.02143	-0.22827
IER2	-0.44042	-0.36143	-1.05063	-0.89723	-0.37511
NR4A2	-0.28288	1.043696	-1.58496	-1.01811	-1.35951
APOE	-1.2103	-1.92688	-0.66658	-0.39203	0.666889
MEST	-0.87536	0.519329	-0.61891	-1.54862	-1.2789
PAM	-0.48106	-0.63294	N/A	-2.00513	-1.58393
JUNB	-0.16794	-0.46411	-1.47275	-1.01022	-0.45578
NR4A1	-0.72993	0.327068	-1.58496	-1.23867	-0.49383
DUSP1	-0.57793	0.233634	-1.25154	-2.09088	-0.73642
PRL	-1.52223	-0.13442	-1.74729	-1.38738	-1.38451
FOS	-0.63639	-0.10355	-1.419	-1.83254	-0.49539
FOSB	-0.52546	-0.45626	-1.98966	-2.1077	-0.65799
HLA-A	-0.73666	-1.91946	0.366782	-1.2991	-4.03562
KLF4	-0.93346	0.502969	-4.05063	-3.04687	-1.13998
HLA-DQB1	-2.37902	-3.21791	-1.77761	-3.14556	-2.22345

CREB1 is a member of the leucine zipper family of DNA binding proteins. It binds to the cAMP-responsive element (CRE) in the promoter region of genes. The threshold for change is Log2FC =±0.58. Yellow highlight indicates genes important for hormone production in pituitary.

Supplemental Table S8. Myc Protooncogene

Genes in the MYC network	Somatotropes	Lactotropes	Thyrotropes	Corticotropes	Sox2 Stem Cells
Hspa1b	N/A	3.164518	1.070389	2.528195	1.024118
HSPB1	-0.31169	2.406888	0.304855	1.27735	0.743395
HSP90AA1	0.529043	0.917232	1.426751	0.467204	0.433861
HSPH1	1.100146	1.410478	1.392317	-0.44908	-0.12873
SERPINH1	N/A	2.093733	N/A	N/A	0.231163
SERPINE1	N/A	1.94581	N/A	N/A	N/A
Mt1	0.281296	-0.35416	0.204358	1.141934	0.660831
RPSA	-0.31214	0.076521	0.91503	0.407298	0.128425
Mt2	1.056448	-0.20348	-0.89308	0.705844	0.78644
GADD45G	-0.04947	0.592755	-0.45568	N/A	0.573699
RPL38	N/A	0.305805	0.774079	0.620785	-0.21567
RPL37	N/A	0.146436	0.460133	0.633259	-0.15788
Calm1	-1.13432	-0.27658	0.969106	0.786982	0.280522
APP	0.20867	-0.39523	1.018859	0.274502	0.646949
MIF	-0.52943	0.164597	-0.38247	0.355989	-0.06268
RHOB	0.559577	1.087781	-0.89308	-1.07469	0.090413
CAMK2N1	-0.54848	0.619582	0.850424	0.302678	-0.79305
ODC1	0.516508	0.050583	0.222392	-0.48543	-0.25998
RPL22L1	-0.47995	-0.32046	0.503963	0.165314	-0.20072
PEG3	N/A	0.933089	0.08153	-0.46616	-0.73294
DBI	-0.49814	0.385532	-0.51457	0.084237	N/A
JUN	0.107065	0.855591	-1.19265	-0.55421	-0.06548
RPL7A	-0.78204	-0.21629	-0.35411	-0.17074	-0.11123
RPS26	-0.59685	-0.55291	0.325949	-0.19725	-0.32499
Rpl23a	-0.8681	-0.67072	0.485427	-0.12686	-0.3682
RHOA	-0.36887	-1.1308	-1.14684	-0.22669	0.233462
KLF6	-0.81593	0.505714	N/A	-2.15269	N/A
DDX21	N/A	N/A	N/A	-1.81166	N/A
SPP1	N/A	N/A	-3.94753	N/A	N/A
COX5A	-1.16031	-1.11342	-0.5502	-0.73765	-0.17729
ZFP36	N/A	-0.57727	-0.34792	-0.94596	0.124612
GLUL	-0.77603	-1.41253	N/A	-0.81166	0.314629
EGR1	-0.22039	N/A	-1.11464	-1.02143	-0.22827
XIST	N/A	0.20516	-1.49033	-2.84728	-0.33867
PAM	-0.48106	-0.63294	N/A	-2.00513	-1.58393
PSMB8	-0.47284	-1.36773	N/A	-1.90476	-1.57619
DUSP1	-0.57793	0.233634	-1.25154	-2.09088	-0.73642
FOS	-0.63639	-0.10355	-1.419	-1.83254	-0.49539
ACTB	-1.22324	-1.4011	-1.77761	-1.17735	-0.36922
ASCL1	-1.85546	0.834041	-1.89308	-0.53412	-3.16687
HLA-E	-0.59796	N/A	N/A	-2.06799	-2.45066
ANGPT1	-1.881	0.42283	-2	-2.39662	-1.3393
HLA-A	-0.73666	-1.91946	0.366782	-1.2991	-4.03562
KLF4	-0.93346	0.502969	-4.05063	-3.04687	-1.13998

Myc is a proto-oncogene, also known as bHLH transcription factor. The threshold for change is Log2FC =±0.58.
Yellow highlight indicates genes important for hormone production in pituitary.

Supplemental Table S9. MLX Interacting Protein					
**Genes in MLXIPL network**	**Somatotropes**	**Lactotropes**	**Thyrotropes**	**Corticotropes**	**Sox2 Stem Cells**
RPL35	0.109184	0.56293	0.916979	0.549139	0.158424
RPS28	-0.35648	0.453345	0.899473	0.752474	N/A
RPS29	-0.05507	0.25663	0.921311	0.664825	0.093659
RPSA	-0.31214	0.076521	0.91503	0.407298	0.128425
RPL38	N/A	0.305805	0.774079	0.620785	-0.21567
RPL37	N/A	0.146436	0.460133	0.633259	-0.15788
RPL37A	-0.3088	N/A	0.864906	0.478687	-0.06226
RPL5	-0.274	0.138417	0.593951	0.152544	-0.19686
RPL4	-0.38718	0.324583	0.222392	0.22582	-0.09288
RPL7	-0.3896	-0.14037	0.492313	0.575001	-0.12873
RPL11	-0.29062	-0.09173	0.379673	0.286447	-0.1073
RPS21	-0.19242	0.104198	0.521953	0.175827	-0.23662
RPS17	N/A	-0.15253	0.918058	0.424102	-0.24361
Rpl36a	-0.58519	-0.0514	0.747766	0.267994	-0.38027
RPS12	-0.40515	-0.19385	0.421977	0.220108	-0.1257
Rps3a1	-0.33826	N/A	0.611604	0.186737	-0.34114
RPS8	-0.41128	-0.14144	0.661504	0.061433	-0.06725
RPL13A	-0.31114	N/A	0.366782	0.497295	-0.19555
RPS10	-0.51277	-0.10911	0.397569	0.138393	-0.08143
RPS19	-0.40627	-0.29783	0.521647	0.445339	-0.11693
RPL27A	-0.29874	-0.06649	0.299068	0.352379	-0.22923
RPL19	-0.3544	-0.10802	0.495769	0.327736	-0.19551
RPS27A	-0.29609	-0.14186	0.444424	0.248002	-0.27143
RPL10	-0.26816	0.050979	0.245319	0.170288	-0.46689
RPL22L1	-0.47995	-0.32046	0.503963	0.165314	-0.20072
RPL21	-0.43335	-0.26124	0.381825	0.139968	-0.31825
RPL17	-0.48042	-0.06532	0.417304	0.121636	-0.39833
RPL9	-0.41941	-0.23119	0.408138	0.255258	-0.11548
RPL23	-0.55424	-0.13791	0.423014	N/A	-0.25441
RPL41	-0.5292	N/A	0.244604	0.182646	-0.30261
Rpl32	-0.5532	-0.14969	0.638336	N/A	-0.52068
RPS20	-0.54673	-0.29514	0.40073	0.189384	-0.27734
RPS7	-0.46603	-0.05832	0.105878	0.116301	-0.4703
RPL30	-0.33149	-0.17102	0.330308	0.09255	-0.50876
RPS14	-0.44192	-0.19622	0.338451	0.276917	-0.36294
RPL26	-0.54757	-0.18673	0.435585	0.1243	-0.28027
RPL24	-0.62339	-0.33127	0.294768	0.193713	-0.3704
RPS23	-0.47873	-0.24984	0.313815	0.110075	-0.42391
RPL7A	-0.78204	-0.21629	-0.35411	-0.17074	-0.11123
RPS26	-0.59685	-0.55291	0.325949	-0.19725	-0.32499
RPL35A	-0.46205	-0.45105	0.051281	0.254497	-0.42907
Rpl23a	-0.8681	-0.67072	0.485427	-0.12686	-0.3682
RPS18	-0.91515	-0.40109	0.086509	-0.22449	-0.74726
MLXIP functions as a part of a heterodimer to activate transcription. Threshold for change is Log2FC =±0.58 Yellow highlight indicates genes important for hormone production in pituitary.					

Supplemental Table S10. Insulin

Genes in the insulin network	Somatotropes	Lactotropes	Thyrotropes	Corticotropes	Sox2 Stem Cells
HSP90AA1	0.529043	0.917232	1.426751	0.467204	0.433861
DNAJB4	1.337185	1.800372	1	0.374758	-0.05834
GH	0.619861	0.541708	0.113522	0.237828	0.585757
HSPA8	0.303237	0.590325	0.649814	0.459727	0.453823
HSPE1	0.291881	0.495002	0.830075	0.813054	0.216516
SERPINE1	N/A	1.94581	N/A	N/A	N/A
AEBP1	N/A	0.945646	0.630477	-0.5162	-0.30227
Mt1	0.281296	-0.35416	0.204358	1.141934	0.660831
ALDOA	-0.18667	0.203056	-0.15417	0.377047	0.167932
HSP90B1	N/A	0.552658	0.222392	-0.13215	0.26101
APP	0.20867	-0.39523	1.018859	0.274502	0.646949
HSPA5	0.299454	0.704754	0.074294	-0.08268	-0.34517
PPIA	-0.51609	-0.5164	0.451874	0.437089	0.397575
MIF	-0.52943	0.164597	-0.38247	0.355989	-0.06268
ODC1	0.516508	0.050583	0.222392	-0.48543	-0.25998
DBI	-0.49814	0.385532	-0.51457	0.084237	N/A
Rpl32	-0.5532	-0.14969	0.638336	N/A	-0.52068
DLK1	0.132952	0.566258	0.657841	-1.07947	-1.48563
RPS14	-0.44192	-0.19622	0.338451	0.276917	-0.36294
JUN	0.107065	0.855591	-1.19265	-0.55421	-0.06548
ATF3	N/A	0.716209	-0.47275	-0.52487	N/A
IGFBP5	-0.7156	-0.99708	1	-0.54862	0.768804
POMC	-0.88829	-1.32076	0.485427	0.523383	N/A
COX4I1	-0.6645	-0.67383	N/A	0.191064	N/A
SOCS3	0.228753	-0.72207	0.052467	-0.94039	0.124247
CEBPB	0.098603	-0.14968	-1.51457	-0.28757	0.262057
RHOA	-0.36887	-1.1308	-1.14684	-0.22669	0.233462
EGR1	-0.22039	N/A	-1.11464	-1.02143	-0.22827
NR4A2	-0.28288	1.043696	-1.58496	-1.01811	-1.35951
APOE	-1.2103	-1.92688	-0.66658	-0.39203	0.666889
JUNB	-0.16794	-0.46411	-1.47275	-1.01022	-0.45578
NR4A1	-0.72993	0.327068	-1.58496	-1.23867	-0.49383
DUSP1	-0.57793	0.233634	-1.25154	-2.09088	-0.73642
PRL	-1.52223	-0.13442	-1.74729	-1.38738	-1.38451
FOS	-0.63639	-0.10355	-1.419	-1.83254	-0.49539
ACTB	-1.22324	-1.4011	-1.77761	-1.17735	-0.36922
FOSB	-0.52546	-0.45626	-1.98966	-2.1077	-0.65799
KLF4	-0.93346	0.502969	-4.05063	-3.04687	-1.13998

Insulin is a pancreatic hormone that regulates serum glucose levels. Threshold for change is Log2FC =±0.58. Yellow highlight indicates genes important for hormone production in pituitary.

Supplemental Table S11 Rictor					
**Genes in the Rictor network**	**Somatotropes**	**Lactotropes**	**Thyrotropes**	**Corticotropes**	**Sox2 Stem Cells**
RPS29	-0.05507	0.25663	0.921311	0.664825	0.093659
RPSA	-0.31214	0.076521	0.91503	0.407298	0.128425
RPL38	N/A	0.305805	0.774079	0.620785	-0.21567
RPL4	-0.38718	0.324583	0.222392	0.22582	-0.09288
RPL7	-0.3896	-0.14037	0.492313	0.575001	-0.12873
RPL11	-0.29062	-0.09173	0.379673	0.286447	-0.1073
RPS21	-0.19242	0.104198	0.521953	0.175827	-0.23662
Rps3a1	-0.33826	N/A	0.611604	0.186737	-0.34114
RPS8	-0.41128	-0.14144	0.661504	0.061433	-0.06725
RPL13A	-0.31114	N/A	0.366782	0.497295	-0.19555
RPS10	-0.51277	-0.10911	0.397569	0.138393	-0.08143
RPS19	-0.40627	-0.29783	0.521647	0.445339	-0.11693
RPS27A	-0.29609	-0.14186	0.444424	0.248002	-0.27143
RPL10	-0.26816	0.050979	0.245319	0.170288	-0.46689
RPL21	-0.43335	-0.26124	0.381825	0.139968	-0.31825
Cox6c	-0.57233	-0.59785	-0.06711	0.272878	0.324376
RPL17	-0.48042	-0.06532	0.417304	0.121636	-0.39833
RPL9	-0.41941	-0.23119	0.408138	0.255258	-0.11548
RPL23	-0.55424	-0.13791	0.423014	N/A	-0.25441
RPL41	-0.5292	N/A	0.244604	0.182646	-0.30261
NDUFS3	-0.49289	-1.38428	0.940621	0.087416	-0.2018
NDUFB3	-0.728	-0.79811	-0.06711	0.238971	N/A
RPL30	-0.33149	-0.17102	0.330308	0.09255	-0.50876
RPL26	-0.54757	-0.18673	0.435585	0.1243	-0.28027
Rpl34	-0.37802	-0.21759	-0.07056	0.0876	-0.60671
COX8A	-0.78789	-0.44187	N/A	0.490103	-0.09311
UQCR11	-1.27561	-0.3511	-0.29698	0.296101	-0.12344
RPS23	-0.47873	-0.24984	0.313815	0.110075	-0.42391
RPL7A	-0.78204	-0.21629	-0.35411	-0.17074	-0.11123
FAU	-0.46454	-0.29972	N/A	0.150837	-0.32758
PSMB3	-1.16834	-0.78619	0.222392	N/A	-0.42091
RPS26	-0.59685	-0.55291	0.325949	-0.19725	-0.32499
COX4I1	-0.6645	-0.67383	N/A	0.191064	N/A
RPL35A	-0.46205	-0.45105	0.051281	0.254497	-0.42907
Rpl23a	-0.8681	-0.67072	0.485427	-0.12686	-0.3682
RPS18	-0.91515	-0.40109	0.086509	-0.22449	-0.74726
COX5A	-1.16031	-1.11342	-0.5502	-0.73765	-0.17729
EGR1	-0.22039	N/A	-1.11464	-1.02143	-0.22827
PSMB8	-0.47284	-1.36773	N/A	-1.90476	-1.57619
Rictor is a component of the mTORC2 protein complex that regulates cell growth, survival, and proliferation. Threshold for change is Log2FC =±0.58					

Supplemental Table S12 Tumor Necrosis Factor (TNF)					
**Genes in the TNF network**	**Somatotropes**	**Lactotropes**	**Thyrotropes**	**Corticotropes**	**Sox2 Stem Cells**
HSPA1A/HSPA1B	1.726227	2.805155	2.321928	2.151819	0.728662
SERPINE1	N/A	1.94581	N/A	N/A	N/A
Mt1	0.281296	-0.35416	0.204358	1.141934	0.660831
PHLDA1	N/A	2.750554	N/A	N/A	-0.29538
RND3	N/A	1.104961	N/A	N/A	-0.19079
RPSA	-0.31214	0.076521	0.91503	0.407298	0.128425
LCN2	N/A	N/A	N/A	N/A	1.959803
HSP90B1	N/A	0.552658	0.222392	-0.13215	0.26101
Mt2	1.056448	-0.20348	-0.89308	0.705844	0.78644
SOX4	0.266795	0.646076	-1.13124	-1.14423	0.21919
ODC1	0.516508	0.050583	0.222392	-0.48543	-0.25998
JUN	0.107065	0.855591	-1.19265	-0.55421	-0.06548
ATF3	N/A	0.716209	-0.47275	-0.52487	N/A
IGFBP5	-0.7156	-0.99708	1	-0.54862	0.768804
ARC	0.345452	1.524964	N/A	-1.48973	0.227707
POMC	-0.88829	-1.32076	0.485427	0.523383	N/A
JUND	-0.13867	-0.0758	-1.21057	-0.58529	-0.05585
SOCS3	0.228753	-0.72207	0.052467	-0.94039	0.124247
CEBPB	0.098603	-0.14968	-1.51457	-0.28757	0.262057
RHOA	-0.36887	-1.1308	-1.14684	-0.22669	0.233462
KLF6	-0.81593	0.505714	N/A	-2.15269	N/A
BTG2	0.192092	0.305083	-1.31294	-0.64273	N/A
SPP1	N/A	N/A	-3.94753	N/A	N/A
ZFP36	N/A	-0.57727	-0.34792	-0.94596	0.124612
CITED2	-0.25756	0.747335	-1.58496	-1.65965	-0.87893
KCNQ1OT1	0.185182	0.324982	-0.33015	-0.64173	-0.30957
GLUL	-0.77603	-1.41253	N/A	-0.81166	0.314629
EGR1	-0.22039	N/A	-1.11464	-1.02143	-0.22827
Iigp1	N/A	N/A	N/A	-1.51209	-3.46
IER2	-0.44042	-0.36143	-1.05063	-0.89723	-0.37511
NR4A2	-0.28288	1.043696	-1.58496	-1.01811	-1.35951
APOE	-1.2103	-1.92688	-0.66658	-0.39203	0.666889
IFITM3	-0.05186	-1.8888	-0.30812	-1.60307	0.232031
NFKBIZ	-0.4789	-0.8823	N/A	-2.28559	-1.24194
RBPMS	-0.41005	-2.42545	N/A	-1.44908	0.417235
JUNB	-0.16794	-0.46411	-1.47275	-1.01022	-0.45578
PSMB8	-0.47284	-1.36773	N/A	-1.90476	-1.57619
NR4A1	-0.72993	0.327068	-1.58496	-1.23867	-0.49383
DUSP1	-0.57793	0.233634	-1.25154	-2.09088	-0.73642
PRL	-1.52223	-0.13442	-1.74729	-1.38738	-1.38451
FOS	-0.63639	-0.10355	-1.419	-1.83254	-0.49539
MIA	-2.53728	-2.75864	-1.28011	-0.65668	0.075029
B2M	-0.6586	-2.02852	-0.13124	-2.86412	-1.27239
HLA-A	-0.73666	-1.91946	0.366782	-1.2991	-4.03562
CD74	-2.54476	-4.95759	-3.32193	-2.69207	-1.99757

TNF is a cytokine that plays a role in cell growth, differentiation and lipid metabolism . Threshold for change is Log2FC =±0.58
Yellow highlight indicates genes important for hormone production in pituitary.

Supplemental Table S13. Epidermal Growth Factor					
**Genes in the Egf network**	**Somatotropes**	**Lactotropes**	**Thyrotropes**	**Corticotropes**	**Sox2 Stem Cells**
GAL	0.915721	1.432169	-0.61891	0.238718	N/A
SERPINE1	N/A	1.94581	N/A	N/A	N/A
PHLDA1	N/A	2.750554	N/A	N/A	-0.29538
LCN2	N/A	N/A	N/A	N/A	1.959803
EEF1A1	-0.10962	0.379952	0.776981	0.350128	-0.13829
RGS2	0.349359	1.227614	N/A	N/A	-0.12407
SOX4	0.266795	0.646076	-1.13124	-1.14423	0.21919
TXNIP	-0.35636	0.647803	0.444785	-0.26232	0.419772
RHOB	0.559577	1.087781	-0.89308	-1.07469	0.090413
ODC1	0.516508	0.050583	0.222392	-0.48543	-0.25998
CHGB	-0.34433	0.709971	1.48224	-0.80658	-1.0678
CGA	-0.84349	-1.26996	0.957144	N/A	N/A
JUN	0.107065	0.855591	-1.19265	-0.55421	-0.06548
ATF3	N/A	0.716209	-0.47275	-0.52487	N/A
IGFBP5	-0.7156	-0.99708	1	-0.54862	0.768804
ARC	0.345452	1.524964	N/A	-1.48973	0.227707
PSMB3	-1.16834	-0.78619	0.222392	N/A	-0.42091
DDX5	-0.38517	-0.10501	-0.3356	-0.50247	N/A
EPCAM	-0.35355	0.065485	N/A	-0.83511	-0.08999
SCG2	-0.51019	N/A	0.846349	-0.3295	-1.42382
JUND	-0.13867	-0.0758	-1.21057	-0.58529	-0.05585
SOCS3	0.228753	-0.72207	0.052467	-0.94039	0.124247
CEBPB	0.098603	-0.14968	-1.51457	-0.28757	0.262057
PLAGL1	-0.48672	0.759865	-0.41504	-0.97671	-2.05834
RHOA	-0.36887	-1.1308	-1.14684	-0.22669	0.233462
BTG2	0.192092	0.305083	-1.31294	-0.64273	N/A
SPP1	N/A	N/A	-3.94753	N/A	N/A
ZFP36	N/A	-0.57727	-0.34792	-0.94596	0.124612
GLUL	-0.77603	-1.41253	N/A	-0.81166	0.314629
EGR1	-0.22039	N/A	-1.11464	-1.02143	-0.22827
IER2	-0.44042	-0.36143	-1.05063	-0.89723	-0.37511
NR4A2	-0.28288	1.043696	-1.58496	-1.01811	-1.35951
ZFP36L2	-0.67308	0.751022	-0.36257	-1.63268	N/A
XIST	N/A	0.20516	-1.49033	-2.84728	-0.33867
JUNB	-0.16794	-0.46411	-1.47275	-1.01022	-0.45578
NR4A1	-0.72993	0.327068	-1.58496	-1.23867	-0.49383
DUSP1	-0.57793	0.233634	-1.25154	-2.09088	-0.73642
PRL	-1.52223	-0.13442	-1.74729	-1.38738	-1.38451
FOS	-0.63639	-0.10355	-1.419	-1.83254	-0.49539
ANGPT1	-1.881	0.42283	-2	-2.39662	-1.3393
FOSB	-0.52546	-0.45626	-1.98966	-2.1077	-0.65799

EGF is a protein that stimulates cell growth and differentiation. Threshold for change is Log2FC =±0.58
Yellow highlight indicates genes important for hormone production in pituitary.

Supplemental Table S14. Ets (E twenty-six) oncogene transcription factor (Elk1)					
Genes in the Elk1 network	Somatotropes	Lactotropes	Thyrotropes	Corticotropes	Sox2 Stem Cells
JUN	0.107065	0.855591	-1.19265	-0.55421	-0.06548
SOCS3	0.228753	-0.72207	0.052467	-0.94039	0.124247
SPP1	N/A	N/A	-3.94753	N/A	N/A
ZFP36	N/A	-0.57727	-0.34792	-0.94596	0.124612
EGR1	-0.22039	N/A	-1.11464	-1.02143	-0.22827
JUNB	-0.16794	-0.46411	-1.47275	-1.01022	-0.45578
PSMB8	-0.47284	-1.36773	N/A	-1.90476	-1.57619
NR4A1	-0.72993	0.327068	-1.58496	-1.23867	-0.49383
PRL	-1.52223	-0.13442	-1.74729	-1.38738	-1.38451
FOS	-0.63639	-0.10355	-1.419	-1.83254	-0.49539
FOSB	-0.52546	-0.45626	-1.98966	-2.1077	-0.65799

Elk1 encodes a transcription factor that plays a role in cell growth, differentiation, survival.
Threshold for change is Log2FC =±0.58. Yellow highlight indicates genes important for hormone production in pituitary.

Supplemental Table S15 Protein Kinase C					
**Genes in the PKC network**	**Somatotropes**	**Lactotropes**	**Thyrotropes**	**Corticotropes**	**Sox2 Stem Cells**
SERPINE1	N/A	1.94581	N/A	N/A	N/A
PHLDA1	N/A	2.750554	N/A	N/A	-0.29538
HSP90B1	N/A	0.552658	0.222392	-0.13215	0.26101
APP	0.20867	-0.39523	1.018859	0.274502	0.646949
HSPA5	0.299454	0.704754	0.074294	-0.08268	-0.34517
RGS2	0.349359	1.227614	N/A	N/A	-0.12407
CGA	-0.84349	-1.26996	0.957144	N/A	N/A
JUN	0.107065	0.855591	-1.19265	-0.55421	-0.06548
ATF3	N/A	0.716209	-0.47275	-0.52487	N/A
KLF6	-0.81593	0.505714	N/A	-2.15269	N/A
SPP1	N/A	N/A	-3.94753	N/A	N/A
POU1F1	-0.16625	0.363494	-0.15612	-1.56654	-1.89569
EGR1	-0.22039	N/A	-1.11464	-1.02143	-0.22827
NR4A2	-0.28288	1.043696	-1.58496	-1.01811	-1.35951
JUNB	-0.16794	-0.46411	-1.47275	-1.01022	-0.45578
NR4A1	-0.72993	0.327068	-1.58496	-1.23867	-0.49383
DUSP1	-0.57793	0.233634	-1.25154	-2.09088	-0.73642
PRL	-1.52223	-0.13442	-1.74729	-1.38738	-1.38451
FOS	-0.63639	-0.10355	-1.419	-1.83254	-0.49539
LHB	-1.32939	-2.08079	-3.78034	-0.50963	-0.68574

PKC is a family of enzymes (kinases) that phosphorylate proteins at serine and threonine residues and is involved in many processes. Threshold for change is Log2FC =±0.58 Yellow highlight indicates genes important for hormone production in pituitary.

Supplemental Table S16. Insulin like growth factor (IGF1)					
**Genes in the Igf1 network**	**Somatotropes**	**Lactotropes**	**Thyrotropes**	**Corticotropes**	**Sox2 Stem Cells**
HSPA1A/HSPA1B	1.726227	2.805155	2.321928	2.151819	0.728662
Hspa1b	N/A	3.164518	1.070389	2.528195	1.024118
GH	0.619861	0.541708	0.113522	0.237828	0.585757
SERPINE1	N/A	1.94581	N/A	N/A	N/A
PHLDA1	N/A	2.750554	N/A	N/A	-0.29538
LCN2	N/A	N/A	N/A	N/A	1.959803
GADD45G	-0.04947	0.592755	-0.45568	N/A	0.573699
APP	0.20867	-0.39523	1.018859	0.274502	0.646949
HSPA5	0.299454	0.704754	0.074294	-0.08268	-0.34517
TXNIP	-0.35636	0.647803	0.444785	-0.26232	0.419772
MIF	-0.52943	0.164597	-0.38247	0.355989	-0.06268
ODC1	0.516508	0.050583	0.222392	-0.48543	-0.25998
JUN	0.107065	0.855591	-1.19265	-0.55421	-0.06548
IGFBP5	-0.7156	-0.99708	1	-0.54862	0.768804
DDX5	-0.38517	-0.10501	-0.3356	-0.50247	N/A
SOCS3	0.228753	-0.72207	0.052467	-0.94039	0.124247
CEBPB	0.098603	-0.14968	-1.51457	-0.28757	0.262057
CHGA	N/A	-0.24043	0.385528	0.057715	-1.20273
KLF6	-0.81593	0.505714	N/A	-2.15269	N/A
BTG2	0.192092	0.305083	-1.31294	-0.64273	N/A
SPP1	N/A	N/A	-3.94753	N/A	N/A
POU1F1	-0.16625	0.363494	-0.15612	-1.56654	-1.89569
C1QA	N/A	-2.0507	N/A	0.072868	N/A
ZFP36	N/A	-0.57727	-0.34792	-0.94596	0.124612
CITED2	-0.25756	0.747335	-1.58496	-1.65965	-0.87893
EGR1	-0.22039	N/A	-1.11464	-1.02143	-0.22827
IER2	-0.44042	-0.36143	-1.05063	-0.89723	-0.37511
ZFP36L2	-0.67308	0.751022	-0.36257	-1.63268	N/A
IFITM3	-0.05186	-1.8888	-0.30812	-1.60307	0.232031
JUNB	-0.16794	-0.46411	-1.47275	-1.01022	-0.45578
PSMB8	-0.47284	-1.36773	N/A	-1.90476	-1.57619
NR4A1	-0.72993	0.327068	-1.58496	-1.23867	-0.49383
DUSP1	-0.57793	0.233634	-1.25154	-2.09088	-0.73642
PRL	-1.52223	-0.13442	-1.74729	-1.38738	-1.38451
FOS	-0.63639	-0.10355	-1.419	-1.83254	-0.49539
FOSB	-0.52546	-0.45626	-1.98966	-2.1077	-0.65799
CD74	-2.54476	-4.95759	-3.32193	-2.69207	-1.99757
Igf1 is a growth factor that promotes growth especially of long bones, mediated by GH. It is produced in the liver. Threshold for change is Log2FC =±0.58. Yellow highlight indicates genes important for hormone production in pituitary.					

Supplemental Table S17 Leptin					
Genes in the Leptin network	Somatotropes	Lactotropes	Thyrotropes	Corticotropes	Sox2 Stem Cells
GH	0.619861	0.541708	0.113522	0.237828	0.585757
GAL	0.915721	1.432169	-0.61891	0.238718	N/A
SERPINE1	N/A	1.94581	N/A	N/A	N/A
ALDOA	-0.18667	0.203056	-0.15417	0.377047	0.167932
GNAS	-0.12969	0.237236	0.434997	0.445945	0.254513
APP	0.20867	-0.39523	1.018859	0.274502	0.646949
HSPA5	0.299454	0.704754	0.074294	-0.08268	-0.34517
PRDX1	-0.3986	-0.65077	-0.05063	N/A	0.752753
DLK1	0.132952	0.566258	0.657841	-1.07947	-1.48563
JUN	0.107065	0.855591	-1.19265	-0.55421	-0.06548
PSMB3	-1.16834	-0.78619	0.222392	N/A	-0.42091
POMC	-0.88829	-1.32076	0.485427	0.523383	N/A
Irgm1	N/A	N/A	N/A	N/A	-2.35755
JUND	-0.13867	-0.0758	-1.21057	-0.58529	-0.05585
SOCS3	0.228753	-0.72207	0.052467	-0.94039	0.124247
CEBPB	0.098603	-0.14968	-1.51457	-0.28757	0.262057
SPP1	N/A	N/A	-3.94753	N/A	N/A
ZFP36	N/A	-0.57727	-0.34792	-0.94596	0.124612
EGR1	-0.22039	N/A	-1.11464	-1.02143	-0.22827
JUNB	-0.16794	-0.46411	-1.47275	-1.01022	-0.45578
PRL	-1.52223	-0.13442	-1.74729	-1.38738	-1.38451
FOS	-0.63639	-0.10355	-1.419	-1.83254	-0.49539
TSHB	-1.33024	-3.22334	2.986394	-1.79958	-1.3632
LHB	-1.32939	-2.08079	-3.78034	-0.50963	-0.68574
Leptin: A cytokine produced by adipocytes, which regulates appetite and is trophic for pituitary cells. Threshold for change is Log2FC =±0.58. Yellow highlight indicates genes important for hormone production in pituitary.					

Supplemental Table S18 CREB Binding Protein					
**Genes in the CREBbp network**	**Somatotropes**	**Lactotropes**	**Thyrotropes**	**Corticotropes**	**Sox2 Stem Cells**
DNAJB4	1.337185	1.800372	1	0.374758	-0.05834
GNAS	-0.12969	0.237236	0.434997	0.445945	0.254513
RGS2	0.349359	1.227614	N/A	N/A	-0.12407
CGA	-0.84349	-1.26996	0.957144	N/A	N/A
JUN	0.107065	0.855591	-1.19265	-0.55421	-0.06548
JUND	-0.13867	-0.0758	-1.21057	-0.58529	-0.05585
SOCS3	0.228753	-0.72207	0.052467	-0.94039	0.124247
RHOA	-0.36887	-1.1308	-1.14684	-0.22669	0.233462
CD81	-0.55085	-0.96003	-0.46113	-0.25817	0.111759
EGR1	-0.22039	N/A	-1.11464	-1.02143	-0.22827
NR4A2	-0.28288	1.043696	-1.58496	-1.01811	-1.35951
ZFP36L2	-0.67308	0.751022	-0.36257	-1.63268	N/A
XIST	N/A	0.20516	-1.49033	-2.84728	-0.33867
PDE10A	-0.57369	1.080129	-1.19265	-1.24461	-2.69642
NR4A1	-0.72993	0.327068	-1.58496	-1.23867	-0.49383
DUSP1	-0.57793	0.233634	-1.25154	-2.09088	-0.73642
PRL	-1.52223	-0.13442	-1.74729	-1.38738	-1.38451
FOS	-0.63639	-0.10355	-1.419	-1.83254	-0.49539
TSHB	-1.33024	-3.22334	2.986394	-1.79958	-1.3632
ANGPT1	-1.881	0.42283	-2	-2.39662	-1.3393
FOSB	-0.52546	-0.45626	-1.98966	-2.1077	-0.65799
MIA	-2.53728	-2.75864	-1.28011	-0.65668	0.075029
B2M	-0.6586	-2.02852	-0.13124	-2.86412	-1.27239
KLF2	-0.49105	-2.39688	-1.65208	-3.35598	-0.34576
CD74	-2.54476	-4.95759	-3.32193	-2.69207	-1.99757
Creb-binding protein regulates cell growth and division, prompting cells to mature and differentiate.<br>Threshold for change is Log2FC =±0.58 Yellow highlight indicates genes important for hormone production in pituitary.					

Supplemental Table S19
Signal Transducer and Activator of Transcription (STAT3)

Genes in the Stat3 network	Somatotropes	Lactotropes	Thyrotropes	Corticotropes	Sox2 Stem Cells
SERPINE1	N/A	1.94581	N/A	N/A	N/A
Mt1	0.281296	-0.35416	0.204358	1.141934	0.660831
PHLDA1	N/A	2.750554	N/A	N/A	-0.29538
LCN2	N/A	N/A	N/A	N/A	1.959803
GADD45G	-0.04947	0.592755	-0.45568	N/A	0.573699
SOX4	0.266795	0.646076	-1.13124	-1.14423	0.21919
JUN	0.107065	0.855591	-1.19265	-0.55421	-0.06548
IGFBP5	-0.7156	-0.99708	1	-0.54862	0.768804
POMC	-0.88829	-1.32076	0.485427	0.523383	N/A
Irgm1	N/A	N/A	N/A	N/A	-2.35755
SOCS3	0.228753	-0.72207	0.052467	-0.94039	0.124247
CEBPB	0.098603	-0.14968	-1.51457	-0.28757	0.262057
PLAGL1	-0.48672	0.759865	-0.41504	-0.97671	-2.05834
SPP1	N/A	N/A	-3.94753	N/A	N/A
ZFP36	N/A	-0.57727	-0.34792	-0.94596	0.124612
EGR1	-0.22039	N/A	-1.11464	-1.02143	-0.22827
NR4A2	-0.28288	1.043696	-1.58496	-1.01811	-1.35951
IFITM3	-0.05186	-1.8888	-0.30812	-1.60307	0.232031
NFKBIZ	-0.4789	-0.8823	N/A	-2.28559	-1.24194
JUNB	-0.16794	-0.46411	-1.47275	-1.01022	-0.45578
PSMB8	-0.47284	-1.36773	N/A	-1.90476	-1.57619
NR4A1	-0.72993	0.327068	-1.58496	-1.23867	-0.49383
DUSP1	-0.57793	0.233634	-1.25154	-2.09088	-0.73642
FOS	-0.63639	-0.10355	-1.419	-1.83254	-0.49539
HLA-A	-0.73666	-1.91946	0.366782	-1.2991	-4.03562
KLF2	-0.49105	-2.39688	-1.65208	-3.35598	-0.34576
KLF4	-0.93346	0.502969	-4.05063	-3.04687	-1.13998
HLA-DQA1	-2.80966	-3.09287	-0.51457	-2.77513	-1.92734
CD74	-2.54476	-4.95759	-3.32193	-2.69207	-1.99757

Stat3 is phosphorylated, as part of the JAK/STAT pathway, forms a dimer and becomes a transcription factor for target genes. Threshold for change is Log2FC =±0.58. Yellow highlight indicates genes important for hormone production in pituitary.

Supplemental Table S20. Transforming Growth Factor Beta-1

Genes in the Tgfb1 network	Somatotropes	Lactotropes	Thyrotropes	Corticotropes	Sox2 Stem Cells
HSPA1A/HSPA1B	1.726227	2.805155	2.321928	2.151819	0.728662
HSPB1	-0.31169	2.406888	0.304855	1.27735	0.743395
HSP90AA1	0.529043	0.917232	1.426751	0.467204	0.433861
DNAJB4	1.337185	1.800372	1	0.374758	-0.05834
DNAJA1	1.137235	1.182629	0.222392	0.284808	0.19127
GAL	0.915721	1.432169	-0.61891	0.238718	N/A
SERPINH1	N/A	2.093733	N/A	N/A	0.231163
SERPINE1	N/A	1.94581	N/A	N/A	N/A
CREB3L1	N/A	1.489836	N/A	N/A	N/A
Calm1	-1.13432	-0.27658	0.969106	0.786982	0.280522
SOX4	0.266795	0.646076	-1.13124	-1.14423	0.21919
JUN	0.107065	0.855591	-1.19265	-0.55421	-0.06548
ATF3	N/A	0.716209	-0.47275	-0.52487	N/A
IGFBP5	-0.7156	-0.99708	1	-0.54862	0.768804
ARC	0.345452	1.524964	N/A	-1.48973	0.227707
POMC	-0.88829	-1.32076	0.485427	0.523383	N/A
DDX5	-0.38517	-0.10501	-0.3356	-0.50247	N/A
JUND	-0.13867	-0.0758	-1.21057	-0.58529	-0.05585
SOCS3	0.228753	-0.72207	0.052467	-0.94039	0.124247
SCG5	-0.38191	0.214737	-0.09843	-0.46943	-1.18787
CEBPB	0.098603	-0.14968	-1.51457	-0.28757	0.262057
GLUL	-0.77603	-1.41253	N/A	-0.81166	0.314629
EGR1	-0.22039	N/A	-1.11464	-1.02143	-0.22827
IER2	-0.44042	-0.36143	-1.05063	-0.89723	-0.37511
NR4A2	-0.28288	1.043696	-1.58496	-1.01811	-1.35951
ZFP36L2	-0.67308	0.751022	-0.36257	-1.63268	N/A
APOE	-1.2103	-1.92688	-0.66658	-0.39203	0.666889
RBPMS	-0.41005	-2.42545	N/A	-1.44908	0.417235
JUNB	-0.16794	-0.46411	-1.47275	-1.01022	-0.45578
NR4A1	-0.72993	0.327068	-1.58496	-1.23867	-0.49383
DUSP1	-0.57793	0.233634	-1.25154	-2.09088	-0.73642
PRL	-1.52223	-0.13442	-1.74729	-1.38738	-1.38451
FOS	-0.63639	-0.10355	-1.419	-1.83254	-0.49539
ANGPT1	-1.881	0.42283	-2	-2.39662	-1.3393
TSIX	N/A	-0.70549	-2.28011	-3.39662	-0.62128
FOSB	-0.52546	-0.45626	-1.98966	-2.1077	-0.65799
KDELR2	-0.71172	-1.11402	-1.92961	-2.0028	-2.09397
B2M	-0.6586	-2.02852	-0.13124	-2.86412	-1.27239
KLF2	-0.49105	-2.39688	-1.65208	-3.35598	-0.34576
KLF4	-0.93346	0.502969	-4.05063	-3.04687	-1.13998
HLA-DQB1	-2.37902	-3.21791	-1.77761	-3.14556	-2.22345
HLA-DQA1	-2.80966	-3.09287	-0.51457	-2.77513	-1.92734
CD74	-2.54476	-4.95759	-3.32193	-2.69207	-1.99757

TGFB1 provides instructions for a protein that regulates cell proliferation, maturation, motility and apoptosis. Threshold for change is Log2FC =±0.58

Yellow highlight indicates genes important for hormone production in pituitary.

Supplemental Table S21 Forkhead box protein O1					
**Genes in the Fox01 network**	**Somatotropes**	**Lactotropes**	**Thyrotropes**	**Corticotropes**	**Sox2 Stem Cells**
HSP90AB1	0.50219	0.723253	0.405256	0.480644	0.598557
SERPINE1	N/A	1.94581	N/A	N/A	N/A
GPX3	-0.13642	0.797672	1.013806	-0.34217	-0.95525
HSPA5	0.299454	0.704754	0.074294	-0.08268	-0.34517
SOX4	0.266795	0.646076	-1.13124	-1.14423	0.21919
TXNIP	-0.35636	0.647803	0.444785	-0.26232	0.419772
JUN	0.107065	0.855591	-1.19265	-0.55421	-0.06548
POMC	-0.88829	-1.32076	0.485427	0.523383	N/A
C1QA	N/A	-2.0507	N/A	0.072868	N/A
CITED2	-0.25756	0.747335	-1.58496	-1.65965	-0.87893
EGR1	-0.22039	N/A	-1.11464	-1.02143	-0.22827
JUNB	-0.16794	-0.46411	-1.47275	-1.01022	-0.45578
PSMB8	-0.47284	-1.36773	N/A	-1.90476	-1.57619
PRL	-1.52223	-0.13442	-1.74729	-1.38738	-1.38451
FOS	-0.63639	-0.10355	-1.419	-1.83254	-0.49539
LHB	-1.32939	-2.08079	-3.78034	-0.50963	-0.68574
ANGPT1	-1.881	0.42283	-2	-2.39662	-1.3393
FOSB	-0.52546	-0.45626	-1.98966	-2.1077	-0.65799
KLF2	-0.49105	-2.39688	-1.65208	-3.35598	-0.34576
FOXO1 is a transcription factor that controls cellular growth, glucose metabolism, stem cell homeostasis. Threshold for change is Log2FC =±0.58. Yellow highlight indicates genes important for hormone production in pituitary.					

Supplemental Table S22 Gonadotropin Releasing hormone					
**Genes in the GnRH network**	**Somatotropes**	**Lactotropes**	**Thyrotropes**	**Corticotropes**	**Sox2 Stem Cells**
GH	0.619861	0.541708	0.113522	0.237828	0.585757
RPSA	-0.31214	0.076521	0.91503	0.407298	0.128425
APP	0.20867	-0.39523	1.018859	0.274502	0.646949
CGA	-0.84349	-1.26996	0.957144	N/A	N/A
JUN	0.107065	0.855591	-1.19265	-0.55421	-0.06548
IGFBP5	-0.7156	-0.99708	1	-0.54862	0.768804
EGR1	-0.22039	N/A	-1.11464	-1.02143	-0.22827
JUNB	-0.16794	-0.46411	-1.47275	-1.01022	-0.45578
PRL	-1.52223	-0.13442	-1.74729	-1.38738	-1.38451
FOS	-0.63639	-0.10355	-1.419	-1.83254	-0.49539
LHB	-1.32939	-2.08079	-3.78034	-0.50963	-0.68574
FOSB	-0.52546	-0.45626	-1.98966	-2.1077	-0.65799
GnRH stimulates the gonadotropes to produce LH and FSH. It also may have a. direct effect on other cell types that bear GnRHR, including Gh and Prl cells. Threshold for change is Log2FC =±0.58. Yellow highlight indicates genes important for hormone production in pituitary.					

Supplemental Table S23 Thyrotropin Releasing hormone					
Genes in the TRH network	Somatotropes	Lactotropes	Thyrotropes	Corticotropes	Sox2 Stem Cells
CGA	-0.84349	-1.26996	0.957144	N/A	N/A
JUN	0.107065	0.855591	-1.19265	-0.55421	-0.06548
POMC	-0.88829	-1.32076	0.485427	0.523383	N/A
JUNB	-0.16794	-0.46411	-1.47275	-1.01022	-0.45578
NR4A1	-0.72993	0.327068	-1.58496	-1.23867	-0.49383
DUSP1	-0.57793	0.233634	-1.25154	-2.09088	-0.73642
PRL	-1.52223	-0.13442	-1.74729	-1.38738	-1.38451
FOS	-0.63639	-0.10355	-1.419	-1.83254	-0.49539
ACTB	-1.22324	-1.4011	-1.77761	-1.17735	-0.36922
TSHB	-1.33024	-3.22334	2.986394	-1.79958	-1.3632

TRH stimulates the Thyrotropes to produce TSH and CGA and lactotropes to produce PRL.
Threshold for change is Log2FC =±0.58. Yellow highlight indicates genes important for hormone production in pituitary.

Supplemental Table S24. Cyclic Adenosine Monophosphate					
Genes in the TRH network	Somatotropes	Lactotropes	Thyrotropes	Corticotropes	Sox2 Stem Cells
SERPINE1	N/A	1.94581	N/A	N/A	N/A
APP	0.20867	-0.39523	1.018859	0.274502	0.646949
TXNIP	-0.35636	0.647803	0.444785	-0.26232	0.419772
RHOB	0.559577	1.087781	-0.89308	-1.07469	0.090413
ODC1	0.516508	0.050583	0.222392	-0.48543	-0.25998
CGA	-0.84349	-1.26996	0.957144	N/A	N/A
JUN	0.107065	0.855591	-1.19265	-0.55421	-0.06548
IGFBP5	-0.7156	-0.99708	1	-0.54862	0.768804
ARC	0.345452	1.524964	N/A	-1.48973	0.227707
POMC	-0.88829	-1.32076	0.485427	0.523383	N/A
EPCAM	-0.35355	0.065485	N/A	-0.83511	-0.08999
SOCS3	0.228753	-0.72207	0.052467	-0.94039	0.124247
CEBPB	0.098603	-0.14968	-1.51457	-0.28757	0.262057
CHGA	N/A	-0.24043	0.385528	0.057715	-1.20273
EGR1	-0.22039	N/A	-1.11464	-1.02143	-0.22827
NR4A2	-0.28288	1.043696	-1.58496	-1.01811	-1.35951
MEST	-0.87536	0.519329	-0.61891	-1.54862	-1.2789
JUNB	-0.16794	-0.46411	-1.47275	-1.01022	-0.45578
NR4A1	-0.72993	0.327068	-1.58496	-1.23867	-0.49383
PRL	-1.52223	-0.13442	-1.74729	-1.38738	-1.38451
FOS	-0.63639	-0.10355	-1.419	-1.83254	-0.49539
TSHB	-1.33024	-3.22334	2.986394	-1.79958	-1.3632
LHB	-1.32939	-2.08079	-3.78034	-0.50963	-0.68574

cAMP is a small molecule that is a second messenger which regulates many biological processes. It is derived from ATP from adenylyl cyclases. Threshold for change is Log2FC =±0.58
Yellow highlight indicates genes important for hormone production in pituitary.
